# Culturing of “Unculturable” Subsurface Microbes: Natural Organic Carbon Source Fuels the Growth of Diverse and Distinct Bacteria From Groundwater

**DOI:** 10.3389/fmicb.2020.610001

**Published:** 2020-12-17

**Authors:** Xiaoqin Wu, Sarah Spencer, Sara Gushgari-Doyle, Mon Oo Yee, Jana Voriskova, Yifan Li, Eric J. Alm, Romy Chakraborty

**Affiliations:** ^1^Department of Ecology, Earth and Environmental Sciences Area, Lawrence Berkeley National Laboratory, Berkeley, CA, United States; ^2^Department of Biological Engineering, Massachusetts Institute of Technology, Cambridge, MA, United States

**Keywords:** natural organic carbon, rarely cultivated, novel, microorganism, groundwater, subsurface, cultivation, isolation

## Abstract

Recovery and cultivation of diverse environmentally-relevant microorganisms from the terrestrial subsurface remain a challenge despite recent advances in modern molecular technology. Here, we applied complex carbon (C) sources, i.e., sediment dissolved organic matter (DOM) and bacterial cell lysate, to enrich groundwater microbial communities for 30 days. As comparisons, we also included enrichments amended with simple C sources including glucose, acetate, benzoate, oleic acid, cellulose, and mixed vitamins. Our results demonstrate that complex C is far more effective in enriching diverse and distinct microorganisms from groundwater than simple C. Simple C enrichments yield significantly lower biodiversity, and are dominated by few phyla (e.g., *Proteobacteria* and *Bacteroidetes*), while microcosms enriched with complex C demonstrate significantly higher biodiversity including phyla that are poorly represented in published culture collections (e.g., *Verrucomicrobia*, *Planctomycetes*, and *Armatimonadetes*). Subsequent isolation from complex C enrichments yielded 228 bacterial isolates representing five phyla, 17 orders, and 56 distinct species, including candidate novel, rarely cultivated, and undescribed organisms. Results from this study will substantially advance cultivation and isolation strategies for recovering diverse and novel subsurface microorganisms. Obtaining axenic representatives of “once-unculturable” microorganisms will enhance our understanding of microbial physiology and function in different biogeochemical niches of terrestrial subsurface ecosystems.

## Introduction

Compared to animal and plant hosts, other non-human environments on Earth such as marine sediment, seawater, soil, and the terrestrial subsurface host prodigious and undescribed microbial populations, as most of them have never been cultured and characterized in the laboratory (Lloyd et al., 2018). In the terrestrial subsurface, it is estimated that there are 2,500 × 10^26^ microbial cells, of which more than 70% belong to uncultured clades, and thus their physiologies and ecological impacts remain largely mysterious (Lloyd et al., 2018). Despite rapid technological advances in modern molecular tools – such as metagenomics, metatranscriptomics, and metaproteomics – for identification of key microbial taxa and critical metabolic processes in a given environment, a complete interpretation of omics-based data is still constrained by the unavailability of reference genomes and physiologically characterized isolates (Giovannoni and Stingl, 2007). Challenges in microbial cultivation/isolation in the laboratory have impeded the ability of microbiologists to fully investigate the roles and function of microbes in terrestrial subsurface ecosystems.

Successful recovery and cultivation of environmental microbes in the laboratory critically depends on appropriate growth media and incubation conditions that best mimic the ecological habitat of the bacteria that includes temperature, moisture, salinity, pH, specific trace metals, and nutrients among others (Overmann, 2013). Enrichment culturing is a common initial step in microbial isolation to select for microorganisms with specific metabolisms within the total microbial population. The choice of organic substrate is of paramount importance in enrichment media composition. Yeast extract and simple organic compounds such as glucose, acetate, lactate, pyruvate, and casamino acids are amended routinely, either as an individual carbon (C) source or as a mixture with the understanding that most microbes utilize these C substrates (Eilers et al., 2000). However, these labile C compounds commonly lead to selective and biased growth of microorganisms with faster growth rates, generally considered as the “weeds” (Wawrik et al., 2005; Cui et al., 2016), and have rarely recovered slow growing yet metabolically active and relevant microbes from the environment (Lok, 2015). Partly for this reason, despite the rapid advances in “omics” technologies, we have still only been able to cultivate less than 2% of microbes on Earth in the laboratory (Overmann et al., 2017; Steen et al., 2019; Martiny, 2020).

Rationally designed growth medium that closely mimics the natural environmental habitats of microorganisms has proven to be an effective strategy in recovering diverse and previously uncultivable organisms from various environments (Connon and Giovannoni, 2002; Kaeberlein et al., 2002; Rappe et al., 2002; Zengler et al., 2002; Henson et al., 2016; Nguyen et al., 2018). Specifically, microorganisms in subsurface are reported to grow optimally in low nutrient availability or oligotrophic conditions (Bartelme et al., 2019). In groundwater, dissolved organic matter (DOM) derived from the adjacent sediment contributes to the available C source for microorganisms. Our previous study shows that sediment DOM contains a myriad of heterogenous organic compounds – mostly recalcitrant C such as lignin-like compounds and a small portion of relatively labile C such as carbohydrate- and protein-like compounds (Wu et al., 2018). Other natural C sources available for microorganisms in groundwater can be from dead, lysed microbial biomass turnover. Despite the potential of these natural C sources for diverse microbial cultivation under laboratory conditions, no research has been reported on the application of sediment DOM or microbial cell lysate for cultivation/isolation of microorganisms from the terrestrial subsurface environment.

In this study, we aim to develop an effective cultivation strategy using naturally occurring complex C to recover diverse, rarely cultivated, and novel bacteria from groundwater collected at the Field Research Center (FRC) in Oak Ridge, TN, United States. Our results show that complex C such as sediment DOM and bacterial cell lysate are much more effective than conventional simple C sources in encouraging the growth of diverse and distinct bacteria from groundwater, providing a platform for the recovery of undiscovered bacteria that constitute “microbial dark matter” in the subsurface. Results from this study will aid in the design of successful cultivation strategies to unlock diverse novel, previously uncultured, or ecologically important subsurface microbes for phenotypic and genomic analysis, which will greatly advance our understanding of microbial physiology, roles, and function in biogeochemical cycles in the terrestrial subsurface.

## Materials and Methods

### Preparation of C Stock Solutions

Glucose, sodium acetate, sodium benzoate, cellulose, oleic acid, vitamins, and thioctic acid were purchased from Sigma-Aldrich (St. Louis, MO). Stock solutions of glucose, sodium acetate, and sodium benzoate were prepared by dissolving the chemical in MilliQ-water (18.2 MΩ·cm, 0.22 μm membrane filtered) at 200, 200, and 50 mM, respectively, followed by filter-sterilization with a filtration system [0.22 μm pore-sized, polyethersulfone (PES), Corning]. Oleic acid and cellulose were added to MilliQ-water at an initial concentration of 50 and 20 g/L, respectively, followed by sterilization using an autoclave. Since oleic acid and cellulose are generally insoluble, their concentrations in water are expressed as initial grams per liter. A stock solution of vitamin B complex and thioctic acid (Supplementary Table S1) was prepared in MilliQ-water according to the recipe reported by Balch et al. (1979), and then filter-sterilized (0.22 μm pore-sized, PES, Corning).

Preparation of bacterial cell lysate solution was modified based on published methods (Simmon et al., 2004; Wang et al., 2015). *Pseudomonas* is the most commonly isolated genus from groundwater at Oak Ridge FRC background sites (our study site). Therefore, a strain of *Pseudomonas* spp. previously isolated in our laboratory from FRC background groundwater was used as the representative strain for this purpose. The isolate was grown in a Luria broth (LB) liquid medium at 30°C aerobically until early stationary phase. A 30 ml aliquot of the culture was harvested, followed by centrifugation at 6,000 *g* for 20 min. The supernatant was removed, and the pellet was washed by MilliQ-water three times before being re-suspended in 10 ml of MilliQ-water. A two-step lysis procedure was used, including autoclaving and sonication in a water bath for 2 h, followed by centrifugation at 6,000 *g* for 20 min. The supernatant was decanted and filtered through a syringe filter (0.2 μm pore-sized, PES, Thermo Scientific). The filtrate was stored at 4°C until use. Total organic C (TOC) content of the filtrate, i.e., cell lysate stock solution, was 2.67 g/L, measured by TOC-5050A Total Organic Carbon Analyzer (Shimadzu, Japan).

The sediment used for DOM extraction was collected from a background well FW305 at Oak Ridge FRC, at a depth of 1.1 m below ground surface. The sediment DOM was extracted according to a method previously developed in our lab (Wu et al., 2018). Briefly, the freeze-dried sediment sample was extracted with Milli-Q water *via* rotary shaking (170 rpm) overnight at 35°C, and then sonicated in a water bath for 2 h. The ratio of water and sediment was 4:1 (w/w). After extraction, the water-sediment slurry was centrifuged at 6,000 *g* for 20 min. The supernatant was decanted and sterilized using a filtration system (0.22 μm pore-sized, PES, Corning). Filtrate containing sediment DOM was freeze-dried, and the lyophilized material was stored at −20°C until use.

### Microcosm Enrichment

The groundwater sample was collected from a background well adjoining the sediment well FW305 at Oak Ridge FRC. The sample was shipped immediately to the lab after collection with ice packs and stored at 4°C for up to 1 week. At the time of sampling, groundwater temperature was measured to be 15.4°C, pH was 6.37, dissolved oxygen (DO) was 1.39 mg/L, TOC was 5.9 mg/L, NO_3_^−^ was 0.34 mg/L, PO_4_^3−^ was less than 3.0 mg/L. The DO in groundwater exceeded 0.5 mg/L, indicating that the groundwater sample’s redox state was oxic (Ohio EPA, http://epa.ohio.gov/Portals/28/documents/gwqcp/redox_ts.pdf).

Microcosm incubation experiments were performed in pre-sterilized 250-ml-flasks, each containing 89 ml of filtered groundwater (0.22 μm pore-sized, PES, Corning) as culture medium, 10 ml of unfiltered groundwater (cell density: 2.1 × 10^6^ cells/ml) as inoculum, and 1 ml of individual C stock solution. For oleic acid and cellulose, the stocks were shaken thoroughly to mix and homogenize the solution before adding to the enrichments. For the sediment DOM-amended group, the lyophilized DOM material was fully dissolved in filtered groundwater at 200 mg/L, and filter-sterilized (0.22 μm pore-sized, PES, Corning). TOC content of the filtrate was measured to be 48.4 mg/L. A 90 ml aliquot of the filtrate (containing sediment DOM) was added with 10 ml of unfiltered groundwater. The organic C content added to the microcosms was designed to be at least five times higher than that in background groundwater (TOC 5.9 mg/L). The final concentrations of substrates in the microcosms are listed in Supplementary Table S2.

An unamended control without any additional C source was included in this study, for which each flask only contained 90 ml of filtered groundwater and 10 ml of unfiltered groundwater. All groups were performed in six replicates. One blank control (without inoculum) was included in each group to monitor potential microbial contamination during incubation. All microcosms were incubated aerobically at 25°C in the dark for up to 30 days, with rotary shaking at 100 rpm. At each sampling time point (days 10, 20, and 30), a 10 ml aliquot of subculture was sampled using a sterile volumetric pipette. Microbes were concentrated by filtration through a membrane filter (0.2 μm pore-sized, PES, 25 mm, Sterlitech Corp.). The filter was then removed from the syringe filter holder and was kept frozen at −80°C until DNA extraction.

### DNA Extraction for Microbial Community Analysis

Before DNA extraction was conducted, the filters were cut into 2-mm-wide stripes using sterile blades and put into DNA extraction tubes provided in PowerMax Soil DNA Isolation Kit (MO BIO Laboratories, Inc., Carlsbad, CA). DNA was extracted following the manufacturer’s protocol, and quantified using the Qubit dsDNA HS Assay Kit (Life Technologies, Eugene, OR) with a Qubit fluorometer (Invitrogen, Eugene, OR). Extracted DNA was stored at −20°C until further processing.

### 16S rRNA Gene Amplicon Library Preparation

For analysis of bacterial community composition, a two-step PCR protocol was performed. In the first step, the 16S rRNA gene of V4 variable region was amplified, and in the second step, Illumina barcodes and adapters for sequencing were added. Extracted DNA from enrichments were each aliquoted into one of three randomized plate layouts in a laminar flow hood.

Before the first step PCR, all samples were subjected to a qPCR at multiple dilutions to determine target dilutions and threshold cycles for the first step. We used 16S rRNA gene primers PE16S_V4_U515_F and PE16S_V4_E786R (Supplementary Table S3). Both 1:1 and 1:10 dilutions of each sample were prepared in duplicate with 0.5X SYBR Green I nucleic acid gel stain (Sigma-Aldrich, St. Louis, MO), plus 280 nM for each primer and the standard reagents in the Phusion High-Fidelity PCR Kit (New England BioLabs, Ipswich, MA). Samples were then cycled under the following qPCR conditions: 98°C at 30 s; 30 cycles of 98°C at 30 s, 52°C at 30 s, and 72°C at 30 s; 4°C hold. Threshold cycles were calculated, and dilutions were prepared to normalize samples and ensure consistent amplification cycles across plates. PCR under the same conditions, minus the SYBR Green, was completed in quadruplicate for each sample, and then quadruplicate sets were pooled and purified with Agencourt AMPure XP Beads according to the manufacturer’s protocol (Beckman Coulter, Brea, CA).

The second step PCR was used to add sample indices and final Illumina adaptors to the 16S rRNA gene amplicons. Reactions were compiled using the Phusion High-Fidelity PCR Kit according to the manufacturer’s instructions, with 420 nM indexing primers PE-III-PCR-F and PE-IV-PCR-R (Supplementary Table S3), and then cycled under the following conditions: 98°C at 30 s; 7 cycles of 98°C at 30 s, 83°C at 30 s, and 72°C at 30 s; 4°C hold. Final libraries were purified with Agencourt AMPure XP Beads according to the manufacturer’s protocol, and then quantified and pooled prior to 2 × 250 paired-end sequencing on an Illumina MiSeq in the MIT BioMicro Center. Data are available on the NCBI database under the accession code PRJNA524696.

### 16S rRNA Gene Amplicon Data Processing and Operational Taxonomic Unit Analysis

Raw reads were quality filtered and clustered into operational taxonomic units (OTUs) primarily with the QIIME software package (Caporaso et al., 2010) using default parameters unless otherwise noted. Paired-end reads were joined with the join_paired_ends.py command, and then barcodes were extracted from the successfully joined reads with the extract_barcodes.py command (and additional parameters -c barcode_in_label, -l 16, -s “#”). Quality filtering was accomplished with split_libraries_fastq.py (-barcode_type 16, -min_per_read_length_fraction 0.40, -q 20, -max_barcode_errors 0, -max_bad_run_length 0, and -phred_offset 33). We checked for the correct forward and reverse primers with a custom script and exported reads with primers removed and length trimmed to 225 bp. Finally, chimeric sequences were removed using identify_chimeric_seqs.py (-m usearch61 and -suppress_usearch61_ref), followed by filter_fasta.py.

After quality filtering, the 16S rRNA gene amplicon sequencing resulted in over 10 million prokaryotic 16S rRNA gene reads, which were clustered into 3,463 OTUs with 97% identity. Only rarefied OTU richness was considered further, in order to compensate for differences in sequencing depth between all samples. The SILVA database classifier was used to assign taxonomy to OTUs with default parameters. No DNA was detected in blank controls, suggesting that microbial contamination was negligible during incubation.

The phylogenetic tree of enriched OTUs was constructed with RAxML, and the visualization was performed using iTOL.^1^

### Bacterial Isolation

The complex C (bacterial cell lysate and sediment DOM)-amended enrichments at each time point were used as inocula for further bacterial isolation. Isolation from simple C enrichments was not pursued. The enrichment inoculum was streaked on a complex C agar plate, prepared using the same medium as corresponding liquid enrichment (cell lysate or sediment DOM) with 1.5% agar (BD Biosciences, United States) and were incubated at 27°C in the dark. We also streaked the enrichment sample on diluted, commercially available culture media (with 1.5% agar), i.e., 1/25 LB, 1/25 tryptic soy broth (TSB), and 1/10 Reasoner’s 2A (R2A), to obtain as many different colonies as possible. Bacterial colonies were streaked again if deemed necessary until single axenic colonies were obtained. All the bacterial isolates were confirmed for growth and maintenance on easily available commercial media (1/25 LB, 1/25 TSB, or 1/10 R2A).

### Species Identification

Genomic DNA of bacterial isolates were extracted using a PureLink Genomic DNA Mini Kit (Invitrogen, United States), following the manufacturer’s protocol. 16S rRNA genes were amplified (initial denaturation step at 98°C for 5 min, followed by 30 cycles at 95°C for 30 s, 50°C for 30 s, and 72°C for 2 min, followed by a final step at 72°C for 3 min) using the eubacterial primers (Galkiewicz and Kellogg, 2008) 27F (AGA GTT TGA TCC TGG CTC AG) and 1492R (ACG GCT ACC TTG TTA CGA CTT) purchased from Integrated DNA Technologies, Inc. (United States). Cleanup of PCR products and DNA sequencing were performed at University of California Berkeley DNA Sequencing Facility. Consensus sequences (1,200–1,400 base pairs) from forward and reverse sequences were generated using Geneious (version 9.1.3) and deposited in Genbank under the access codes listed in Supplementary Table S4. The SILVA database^2^ was used for bacterial isolate classification. Sequence alignments, finding nearest neighbors, and phylogenetic tree reconstructions were performed in SILVA using SINA (v1.2.11) and RAxML (Stamatakis, 2014).

### Data Analysis and Statistics

Shannon’s diversity index (*H'*) and multivariate statistics were performed using the R package *vegan*.^3^ OTU distributions were transformed into relative abundances using the function *decostand*. These were subjected to Hellinger transformation before calculation of Bray-Curtis dissimilarity matrices comparing community composition between samples. Nonmetric multidimensional scaling (NMDS) using function *metaMDS* was performed using these dissimilarity matrices. A multivariate ANOVA (MANOVA) model was implemented in the *vegan* function *adonis*. Analysis of similarity (ANOSIM) was carried out based on Bray-Curtis dissimilarities to evaluate the effect of C source and incubation time on community structure. We compared the relative abundance of taxa among the samples and selected enriched OTUs using R software. Samples were compared by one-way ANOVA followed by the Dunnett’s test (*p* < 0.01) for multiple comparisons.

## Results

In this study, we applied a two-step workflow for cultivating and isolating a broad diversity of bacteria from groundwater. Microcosm enrichments amended with different C sources were used as the first step to enrich bacterial species from Oak Ridge FRC groundwater under aerobic condition. We evaluated two types of complex C: sediment DOM and bacterial cell lysate. The bacterial cell lysate was prepared using a native, naturally abundant bacterial strain isolated from FRC groundwater to mimic the cell lysis products available for groundwater microorganisms. For comparison, we also evaluated six simple C sources, i.e., conventional C source (glucose and acetate), naturally occurring compounds (benzoate, oleic acid, and cellulose), and mixed vitamins. The mixed vitamins were included because they are often added as supplements to bacterial growth media (Balch et al., 1979; Coates et al., 1995), and we wanted to test whether they are a limiting factor for support of microbial growth in this experiment. After enrichment cultivation, conventional direct plating was conducted to obtain axenic bacterial isolates from enrichments amended with complex C (i.e., sediment DOM or bacterial cell lysate).

### Natural Complex C Increases Bacterial Diversity in Enrichments

**Figure 1:**
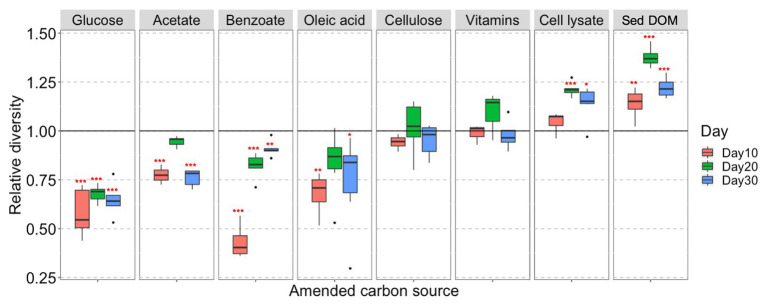
Non-metric multidimensional scaling (NMDS) plot based on Bray-Curtis dissimilarities of bacterial community composition in different C-amended enrichments.

Our results show that both C type and length of incubation have significant influence on bacterial community structure in enrichment cultures. Statistical analysis reveals that C type is the major driver of community dissimilarity (MANOVA/*adonis*, *R*^2^ = 0.56; ANOSIM, *R* = 0.88, *p* = 0.001), with incubation time contributing to a lesser extent to variation (MANOVA/*adonis*, *R*^2^ = 0.09; ANOSIM, *R* = 0.12, *p* = 0.001). We accordingly grouped samples by NMDS ordination based on the type of amended C source (Figure 1). The bacterial community in enrichments amended with glucose, acetate, benzoate, oleic acid, bacterial cell lysate, or sediment DOM clearly differs from the unamended control. The bacterial community composition in cultures amended with simple C (glucose, acetate, benzoate, or oleic acid) are noticeably similar to each other at an early stage of incubation, and then diversify at later stages – while bacterial community composition in cultures amended with complex C (sediment DOM or bacterial cell lysate) separate far from other groups from early on.

**Figure 2:**
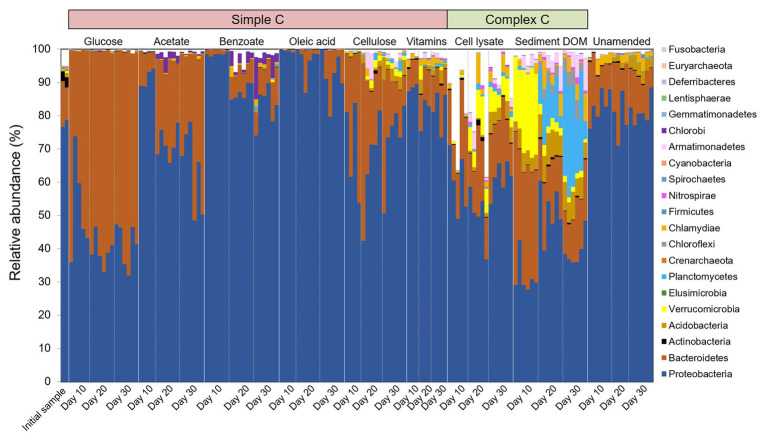
Box and whisker plots illustrating the relative diversity of bacterial community in different C-amended enrichments as compared to the unamended control. Relative diversity is calculated as *H_i_’*/*H_0_’*. *H_i_’* is Shannon’s diversity index of individual sample, *H_0_’* is the average Shannon’s diversity index of unamended control at corresponding sampling time point. Significant difference between the C-amended enrichments and the unamended control is indicated by ^***^ when *p* < 0.001, ^**^ when *p* < 0.01, and ^*^ when *p* < 0.05.

We also observe that the biodiversity of microcosm enrichment is related to the complexity of amended C substrates. The Shannon’s diversity indexes (alpha diversity) for enrichments amended with simple C (glucose, acetate, benzoate, or oleic acid) are generally lower than those in unamended control (Figure 2). This strongly suggests that providing microbial communities with a simple, small organic substrate in growth media will decrease diversity and lead to enrichment of a select few bacterial species that preferentially utilize this substrate. In contrast, the Shannon’s diversity indexes for enrichments amended with complex C (sediment DOM or bacterial cell lysate) are significantly higher than those in unamended control, demonstrating the potential of natural complex C in promoting growth of diverse bacteria (Figure 2).

Cellulose and mixed vitamins show relatively little influence on bacterial community composition and diversity in comparison with the unamended control (Figures 1, 2), and therefore are not included in our further statistical analysis.

### Natural Complex C Enriches Distinct Bacterial Taxa

**Figure 3:**
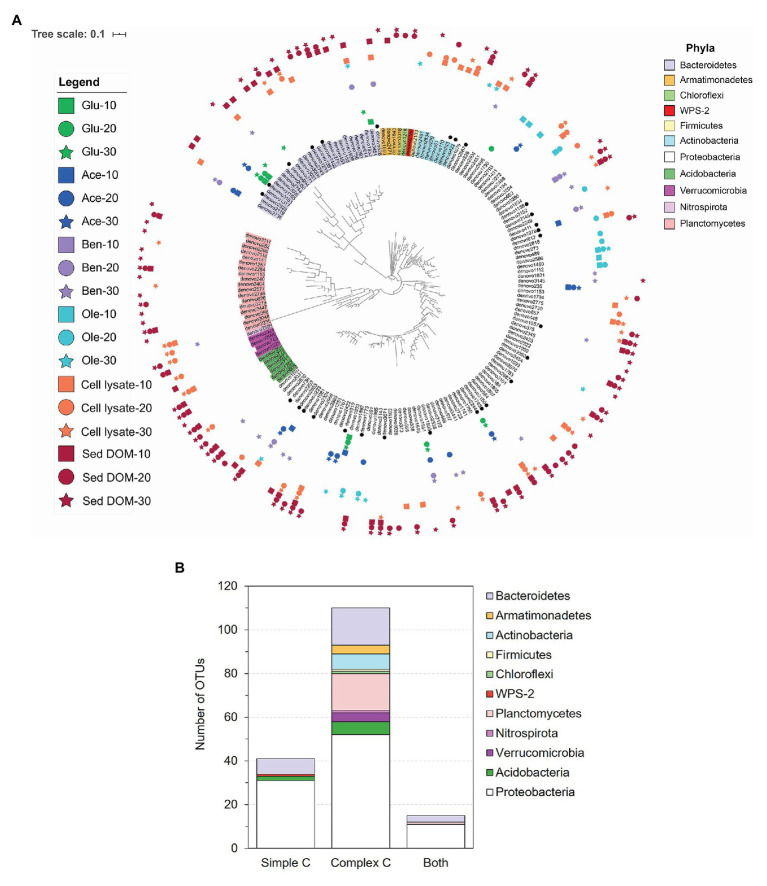
Temporal community structures of the initial groundwater sample and different C-amended enrichments reported as relative abundance of taxonomic phyla over three sampling timepoints (Days 10, 20, and 30). Bar plots that do not sum to 100% indicate the remaining OTUs did not affiliate with a known phylum per SILVA taxonomic classification.

We investigate the short-term response of bacterial community structure to different C sources in enrichment cultures *via* 16S rRNA gene survey. Out of the quality-filtered reads, organisms from 21 phyla and 94 orders are taxonomically identified, which comprise 71–100% of reads, with the exception of two samples (57 and 60%) in the bacterial cell lysate-amended group. The taxonomically identified phyla and abundant orders (with relative abundance > 1% in any sample) are presented in Figure 3 and Supplementary Figure S1, respectively. *Proteobacteria* and *Bacteroidetes* are the two most dominant phyla in all samples. Complex C-amended enrichments contain highly diverse and quite distinct phyla compared to the initial groundwater sample and simple C-amended enrichments (Figure 3). It is worth noting that the rarely cultivated phyla *Verrucomicrobia*, *Planctomycetes*, and *Armatimonadetes*, which are low-abundant taxa (<0.5% at phylum level) in initial sample, are enriched abundantly in sediment DOM-amended cultures with clear succession patterns. *Verrucomicrobia* is enriched at early stages, and then diminishes over time, with relative abundance decreasing from 18–27% at Day 10 to less than 2% at Day 30. Meanwhile, *Planctomycetes* becomes one of the major phyla at later stages, with relative abundance increasing from 0.1–1% at Day 10 to 5–33% at Day 30. *Armatimonadetes* also increases during the incubation period, with relative abundance up to 10% at Day 30.

In microcosms amended with simple C (glucose, acetate, benzoate, or oleic acid), only a few taxonomic orders such as *Caulobacterales*, *Burkholderiales*, *Rhodocyclales*, and *Cytophagales* are enriched, while in complex C-amended microcosms, diverse taxonomic orders are enriched, including those scarcely enriched in other groups, e.g., *Sphingobacteriales*, *Gemmatales*, *Planctomycetales*, *Verrucomicrobiales*, and *Solibacterales* (Supplementary Figure S1).

**Figure 4:**
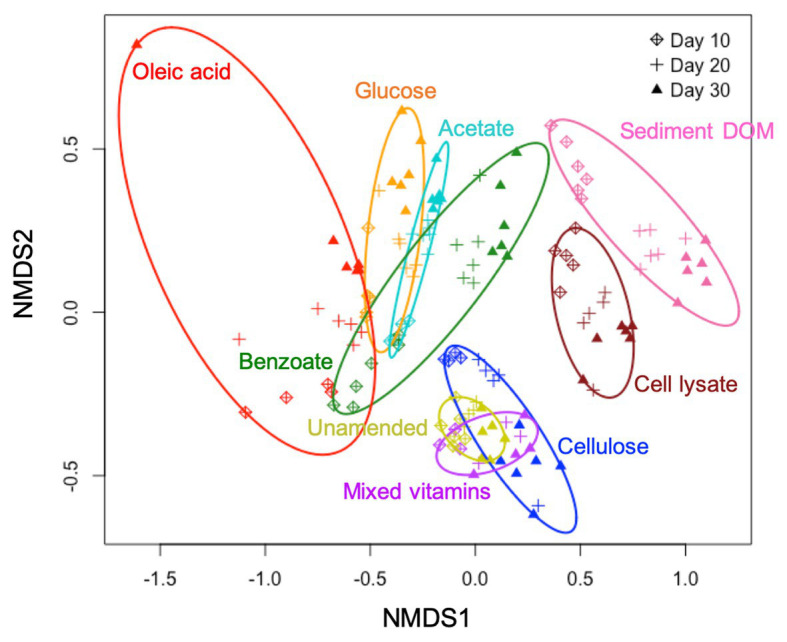
Selected operational taxonomic units (OTUs; one-way ANOVA with Dunnett’s multiple comparison adjustment, *p* < 0.01) that were enriched by simple C (glucose, acetate, benzoate, or oleic acid) or complex C [bacterial cell lysate or sediment dissolved organic matter (DOM)] sources as compared to the unamended control. **(A)** Phylogenetic tree of the 166 enriched OTUs. OTU labels are highlighted according to their phyla. Black dots designate OTUs for which we obtained representative isolates (99–100% identity). Markers surrounding the tree denote the day (10 – square, 20 – circle, or 30 – star) and C substrate in which the OTU was significantly enriched. **(B)** The number of OTUs that were enriched exclusively by simple C, exclusively by complex C, and by both simple and complex C.

Based on one-way ANOVA with Dunnett’s test results, we selected a total of 166 OTUs that were enriched by simple or complex C sources, with significantly (*p* < 0.01) increased relative abundances in the C-amended enrichments compared to the corresponding unamended control at each time point (Figure 4A; Supplementary Table S5). Compared to simple C (glucose, acetate, benzoate, and oleic acid), the complex C (sediment DOM and bacterial cell lysate) show a great advantage in promoting the growth of diverse and distinct bacterial species (Figure 4A). Most of enriched OTUs (110 out of 166) are exclusively enriched by complex C (Figure 4B), especially those from rarely cultivated phyla *Verrucomicrobia*, *Planctomycetes*, and *Armatimonadetes*. A small portion (41 out of 166) is exclusively enriched by simple C, most of which are from the phyla *Proteobacteria* and *Bacteroidetes*, and a few from *Acidobacteria* and WPS-2 (Figure 4B). There are 15 OTUs that can be enriched by both simple and complex C, suggesting that they likely harbor the metabolic potential for utilizing diverse C sources, from simple C to complex C.

Besides amended C source, incubation time also affects the enriched bacterial species. In those 110 OTUs exclusively enriched by complex C, we observe slow growers (25 out of 110) that exhibit significant enrichment at late incubation stage (on Day 30), and also consistent growers (29 out of 110) which were consistently enriched in the cultures from Day 10 to 30 (Figure 4A).

### Novel Bacterial Isolates From Complex C-Amended Enrichments

**Figure 5:**
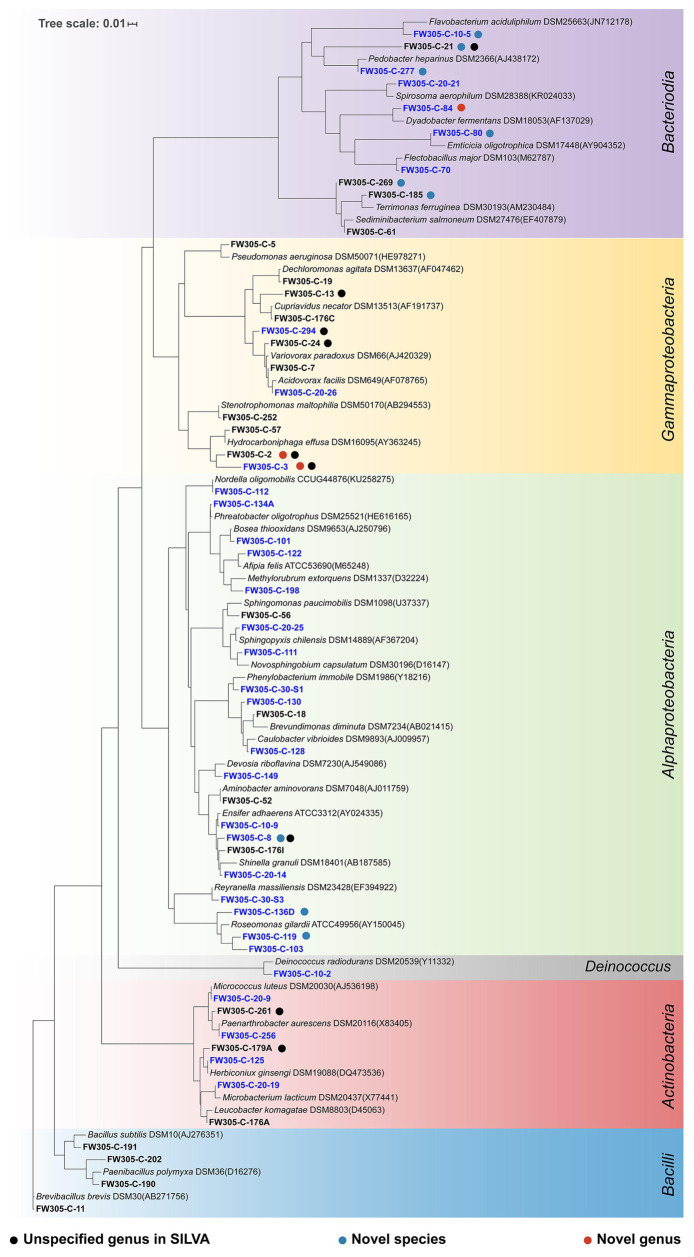
Phylogenetic tree of representative isolates representing 56 distinct bacterial species. The tree is constructed from near full-length 16S rRNA gene sequences. Unspecified genus in SILVA (black), novel candidate species (blue), and novel candidate genus (red) are shown by dot colors. The fonts in blue are those isolated from sediment DOM-amended enrichments and those in black from cell lysate-amended enrichments.

Since complex C (sediment DOM and bacterial cell lysate) shows great potential in enriching diverse and distinct bacterial species, we then used the complex C-amended enrichments as inocula for further isolation. In this study, we obtained a total of 228 bacterial isolates (Supplementary Table S4) representing five phyla, 17 orders, and 56 distinct species (Figure 5; Supplementary Table S6). Our isolates represent both abundant (3–5% relative abundance at OTU level) and rare (<0.01%) species from the initial groundwater sample (Supplementary Table S6).

A comparison between the enrichment and isolation results shows that our bacterial isolates represent 10 out of the 33 enriched orders on all C sources (Supplementary Figure S1), and 28 out of the 166 enriched OTUs (Figure 4A; Supplementary Table S5). We obtained representative isolates not only for the OTUs that are exclusively enriched by complex C, but also for the OTUs that are exclusively enriched by simple C (Figure 4A; Supplementary Table S5). We also obtained bacterial isolates representing slow growers that were only enriched on Day30 (e.g., *denovo*3150) and consistent growers (e.g., *denovo*422, *denovo*1156, and *denovo*2687) from our isolation efforts (Figure 4A; Supplementary Table S5).

**Figure 6:**
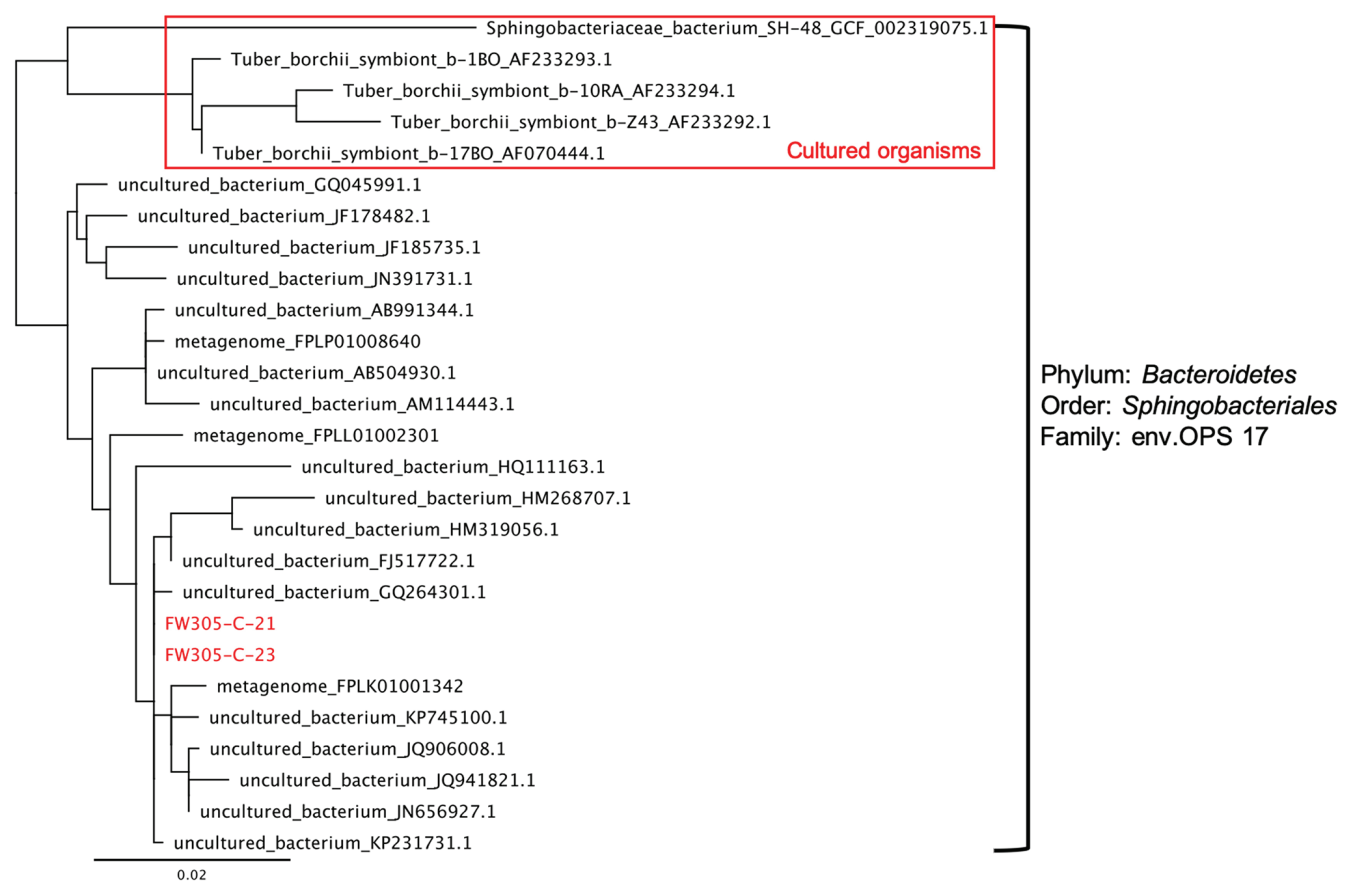
Phylogenetic tree of the isolates FW305-C-21, FW305-C-23, and the most similar bacteria based on 16S rRNA genes. Scale bar indicates a change of 0.02 per nucleotide. The 16S rRNA gene sequences were aligned using SINA against the SILVA alignment, and the maximum likelihood tree was calculated using RAxML. The top five organisms are as far cultured organisms within this family.

The thresholds for determining the novelty of an isolate based on 16S rRNA gene sequence similarity differ slightly in different reports (Stackebrandt and Ebers, 2006; Rossi-Tamisier et al., 2015). Here, we apply the thresholds of 98% for novel species, 95% for novel genera, and 90% for novel families (Nguyen et al., 2018). According to these criteria, of the 56 distinct bacterial species isolated from FRC groundwater, nine belong to candidate novel species and three belong to candidate novel genera (Figure 5; Supplementary Table S6). These novel isolates distribute across two phyla *Proteobacteria* and *Bacteroidetes*, which are the most dominant phyla in the original FRC groundwater sample (data not shown) as well as enrichment cultures in this study. Besides, there are nine undescribed species unassigned at the genus level in the SILVA database, indicating that they are from the less characterized genera with unresolved taxonomy. The reconstructed phylogenetic trees for these novel and undescribed organisms are presented in Figure 6 and Supplementary Figure S2.

## Discussion

There is a compelling need for improving the recovery of diverse bacteria from environments. Several ongoing efforts include modification of growth media/conditions (Vartoukian et al., 2010), use of diluted medium or serial dilution culture (Janssen et al., 2002; Schoenborn et al., 2004), and cultivation with physical separation (e.g., iChip, Nichols et al., 2010 or diffusion chambers, Kaeberlein et al., 2002; Bollmann et al., 2007, 2010). However, the collective capability for recovering microorganisms from the terrestrial subsurface, especially those mediating critical biogeochemical cycles, is still limited. This bottleneck continues to hinder a thorough investigation of microbial ecology and understanding of physiology and true metabolic potential of key organisms residing in terrestrial subsurface ecosystems.

In this study, we demonstrate that natural complex C fuels the growth of much more diverse and distinct groups of microbes compared to traditional simple C sources. Our previous investigation of water-extractable sediment DOM from the same well (FW305), applying ultrahigh resolution mass spectrometry for chemical characterization, shows that sediment DOM is a mixture of heterogeneous naturally occurring substrates, containing lignin, lipids, proteins, tannins, carbohydrates, amino sugars, condensed aromatics, and many uncharacterized compounds (Wu et al., 2018). Besides various C substrates, sediment-derived DOM may contain trace uncharacterized nutrients that potentially benefit the growth of bacteria, contributing to its outperformance in enriching diverse bacteria compared to simple C sources in this study. The complex composition of sediment DOM makes it a suitable source for encouraging the growth of diverse bacterial representatives of *in-situ* environmental communities including those not typically cultivated in the laboratory.

As shown in our enrichment results, almost all species from the rarely cultured phyla *Verrucomicrobia*, *Planctomycetes*, and *Armatimonadetes* show exclusive preference for complex C (Figures 3, 4). Phylum *Verrucomicrobia* was enriched in both cell lysate- and sediment DOM-amended cultures. However, while orders *Pedosphaerales* and *Opitutales* were enriched in bacterial cell lysate-amended cultures at late incubation stage, order *Verrucomicrobiales* was highly enriched in sediment DOM-amended cultures at early incubation stage (Supplementary Figure S1). *Verrucomicrobiales* are known to degrade recalcitrant compounds in different ecosystems (Liu et al., 2020), and genomic analysis of *Verrucomicrobiales* suggests that the type of *Verrucomicrobia* varied among different sites, largely due to differences in complex carbon profile (He et al., 2017). To date, only a handful of *Verrucomicrobia* isolates have been successfully cultivated (Janssen et al., 1997, 2002; Joseph et al., 2003; Matsuzawa et al., 2010; Erikstad and Birkeland, 2015; Spring et al., 2015), although members of this bacterial phylum are highly prevalent in the environment (Bergmann et al., 2011; Freitas et al., 2012). Results from this study may provide clues for designing efficient growth media for cultivating *Verrucomicrobia* from the environment.

The ubiquitous *Planctomycetes* are of deep interest to microbiologists because of their unique characteristics such as their important roles in the global carbon and nitrogen cycles (Wiegand et al., 2018), and their biotechnological relevance for wastewater treatment (Peeters and van Niftrik, 2019). So far only a small portion (~ 2%) of strains in *Planctomycetes* have been isolated in pure cultures (Lage and Bondoso, 2012). Very recently, Wiegand et al. (2020) reported isolation of 79 planctomycetes from different aquatic environments. In our study, we observe that *Planctomycetes* were enriched in sediment-DOM amended incubations over time (Figures 3, 4A). Previous reports suggest that these groups of microbes have a low demand for carbon and nitrogen (Lage and Bondoso, 2012), and are often associated with environments abundant in chitin (Dedysh and Ivanova, 2019). Chitin is commonly present in natural environments including in aquatic sediment. It is a polymer of N-acetylglucosamine, and a structural element of fungi, protozoa, and other small life forms. It is therefore not surprising that *Planctomycetes* comprised up to 33% of the microbial community by Day 30 after the fast growers consumed most of labile C and nutrients in these sediment-derived DOM (which likely contained chitin monomers) incubations (Figure 3). The phylum *Armatimonadetes* lacked an isolated representative until 2011 (Tamaki et al., 2011), and so far, only a few cultivated strains in this phylum have been reported (Lee et al., 2011; Tamaki et al., 2011; Im et al., 2012; Li et al., 2019). The slow growth of *Planctomycetes* and *Armatimonadetes* observed in this study also suggests that they might possess metabolic potential of utilizing relatively recalcitrant DOM, therefore avoid competition for labile DOM with competitive fast growers.

Applying the two-step cultivation strategy, i.e., enrichment followed by isolation, we obtained pure cultures of 56 distinct bacterial species from groundwater, some of which are novel, previously uncultured, and uncharacterized organisms. Although we have not yet isolated pure bacterial strains from *Verrucomicrobia*, *Planctomycetes*, and *Armatimonadetes* in this study, we abundantly enriched them in complex C-amended cultures as comparison to the initial sample, which may augment our ongoing efforts to obtain pure isolates from these phyla in the near future. Our results, as well as other recent reports suggesting growth of rare taxa on gellan gum (Wiegand et al., 2020), will inform our ongoing method development for isolating “unculturable” microbes in the future.

Notably, we obtained isolates (FW305-C-21 and FW305-C-23) from the candidate family env.OPS 17, which is a poorly described family in literature and lacks representative isolates. To date, there are only five described cultured organisms within this family found to be associated with ascomycetous ectomycorrhizal fungi (Barbieri et al., 2000) or in freshwater springs (NCBI database). Our isolates FW305-C-21 and FW305-C-23 are distinct from those five cultured organisms (Figure 6). While several of their close neighbors have been detected *via* molecular tools in various environments including pit (Field et al., 2010), drinking water (Shi et al., 2013), uranium mining wastes (Geissler and Selenska-Pobell, 2005), freshwater lake, pond, soil, and sludge (information from the NCBI database), our isolates are the very first cultured organisms in this distinct clade. Our enrichment results show that species from this candidate family env.OPS 17, i.e., *denovo*1405 (with representative isolate FW305-C-21 and FW305-C-23) and *denovo*2797, exclusively prefer complex C sources (Figure 4A; Supplementary Table S5), which may explain why these organisms have rarely been cultivated in the laboratory.

We also obtained pure cultures of three distinct species: FW305-C-2, FW305-C-3, and FW305-C-57, from an undercharacterized order *Salinisphaerales* which has only 17 reported genomes so far, the second-fewest in the class *Gammaproteobacteria* (NCBI lifemap). The isolates FW305-C-2 and FW305-C-3 are novel candidate genus members (Figure 5; Supplementary Table S6). Phylogenetic analysis of the isolate FW305-C-3 shows that it is close to the genus *Fontimonas* (Supplementary Figure S2). The isolate FW305-C-2 clusters together in the phylogenetic tree with multiple uncultured organisms (Supplementary Figure S2). The only cultured organism in this clade, *Sinobacteraceae* bacterium MG649968.1, was reported very recently from surface freshwater (Sheu et al., 2019). We have therefore made a good contribution to the number of representative cultured organisms in this distinct clade.

In summary, this study demonstrates the potential of natural complex C, especially DOM, for enriching diverse and ecologically relevant bacterial taxa, and for retrieving pure cultures of novel, previously uncultured organisms from the terrestrial subsurface. Our cultivation strategy will benefit future development of effective and ecologically relevant cultivation/isolation strategies. These improved capabilities will be crucial for further understanding of bacterial physiology, functions, and roles in biogeochemical cycles in terrestrial subsurface ecosystems.

## Data Availability Statement

The datasets presented in this study can be found in online repositories. The names of the repository/repositories and accession number(s) can be found in the article/Supplementary Material.

## Author Contributions

XW and RC designed and managed the study. XW, SS, SG-D, MY, JV, and YL generated and analyzed the data. XW, SS, SG-D, MY, EA, and RC prepared and wrote the manuscript. All authors contributed to the article and approved the submitted version.

### Conflict of Interest

The authors declare that the research was conducted in the absence of any commercial or financial relationships that could be construed as a potential conflict of interest.

## Footnotes

^1^https://itol.embl.de

^2^https://www.arb-silva.de/

^3^https://github.com/vegandevs/vegan/
