# Supplementary material for: Culturing of “Unculturable” Subsurface Microbes: Natural Organic Carbon Source Fuels the Growth of Diverse and Distinct Bacteria From Groundwater
Source: Front Microbiol. 2020 Dec 17;11:610001. doi: 10.3389/fmicb.2020.610001 (PMC7773641; doi:10.3389/fmicb.2020.610001)
Supplement: Supplementary file 1 [file Data_Sheet_1.pdf]

Supplementary Information

Culturing of 'Unculturable' Subsurface Microbes: Natural Organic Carbon Source Fuels the Growth of Diverse and Distinct Bacteria from Groundwater

Xiaoqin Wu[1], Sarah Spencer[2], Sara Gushgari-Doyle[1], Mon Oo Yee[1], Jana Voriskova[1], Yifan Li[1], Eric J. Alm[2], Romy Chakraborty[1*]

[1]Department of Ecology, Earth and Environmental Sciences Area, Lawrence Berkeley National Laboratory, Berkeley, California 94720, USA
[2]Department of Biological Engineering, Massachusetts Institute of Technology, Cambridge, Massachusetts 02139, USA

*Corresponding author:
Romy Chakraborty
Address: 70A-3317F, 1 Cyclotron Rd., Berkeley, CA 94720
Tel: (510) 486-4091
Email: rchakraborty@lbl.gov

Figure S1. Relative abundance of enriched taxonomic order (>1% in any sample). Orders having representative isolates in this study are marked with red underlines.

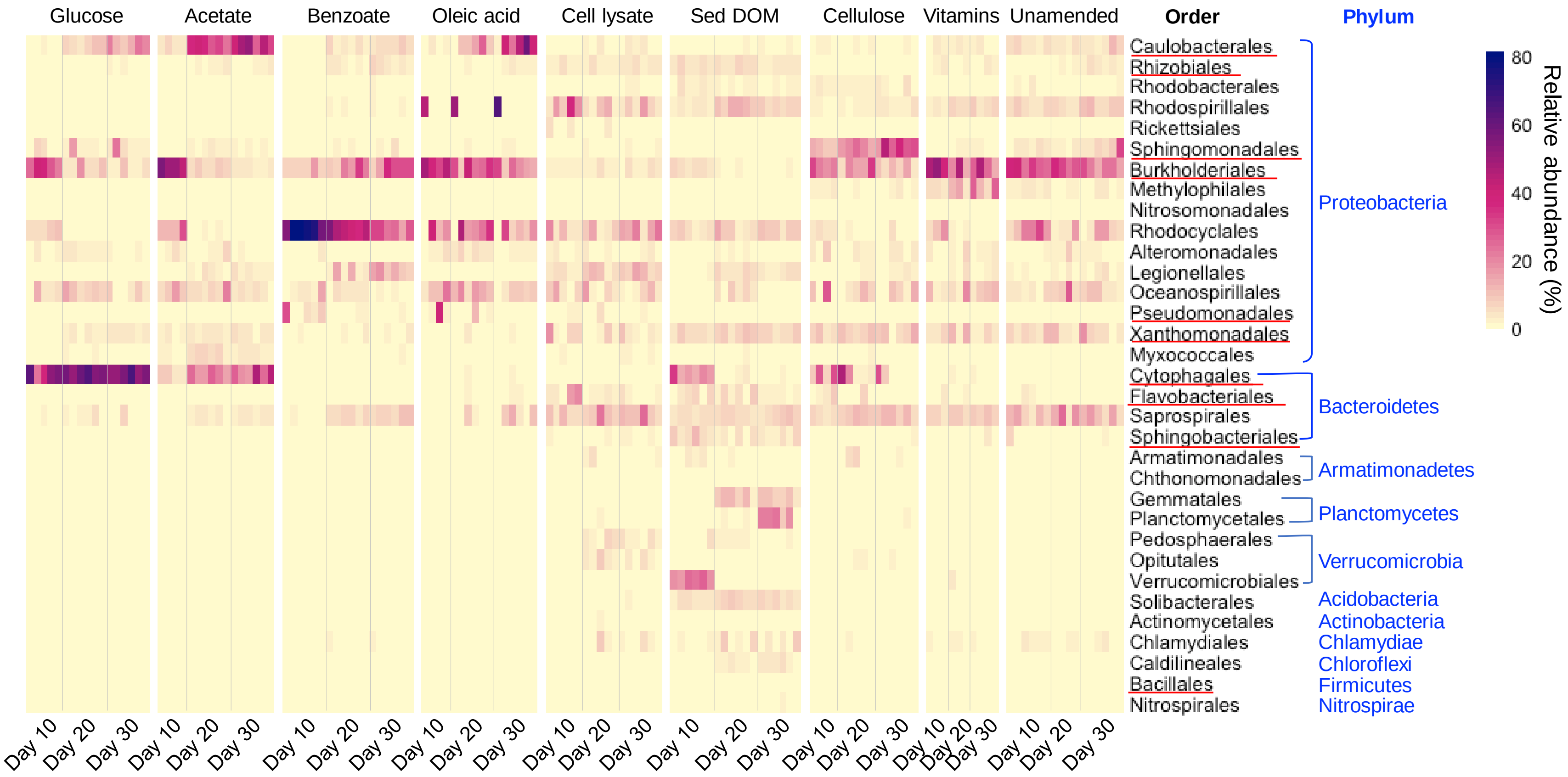

Figure S2. Phylogenetic trees of selected isolates from candidate novel genera/species and undescribed species, and the most similar bacteria based on 16S rRNA genes. Scale bar indicates a change of a certain number per nucleotide. The 16S rRNA gene sequences were aligned using SINA against the SILVA alignment and the maximum likelihood tree was calculated using RAxML.

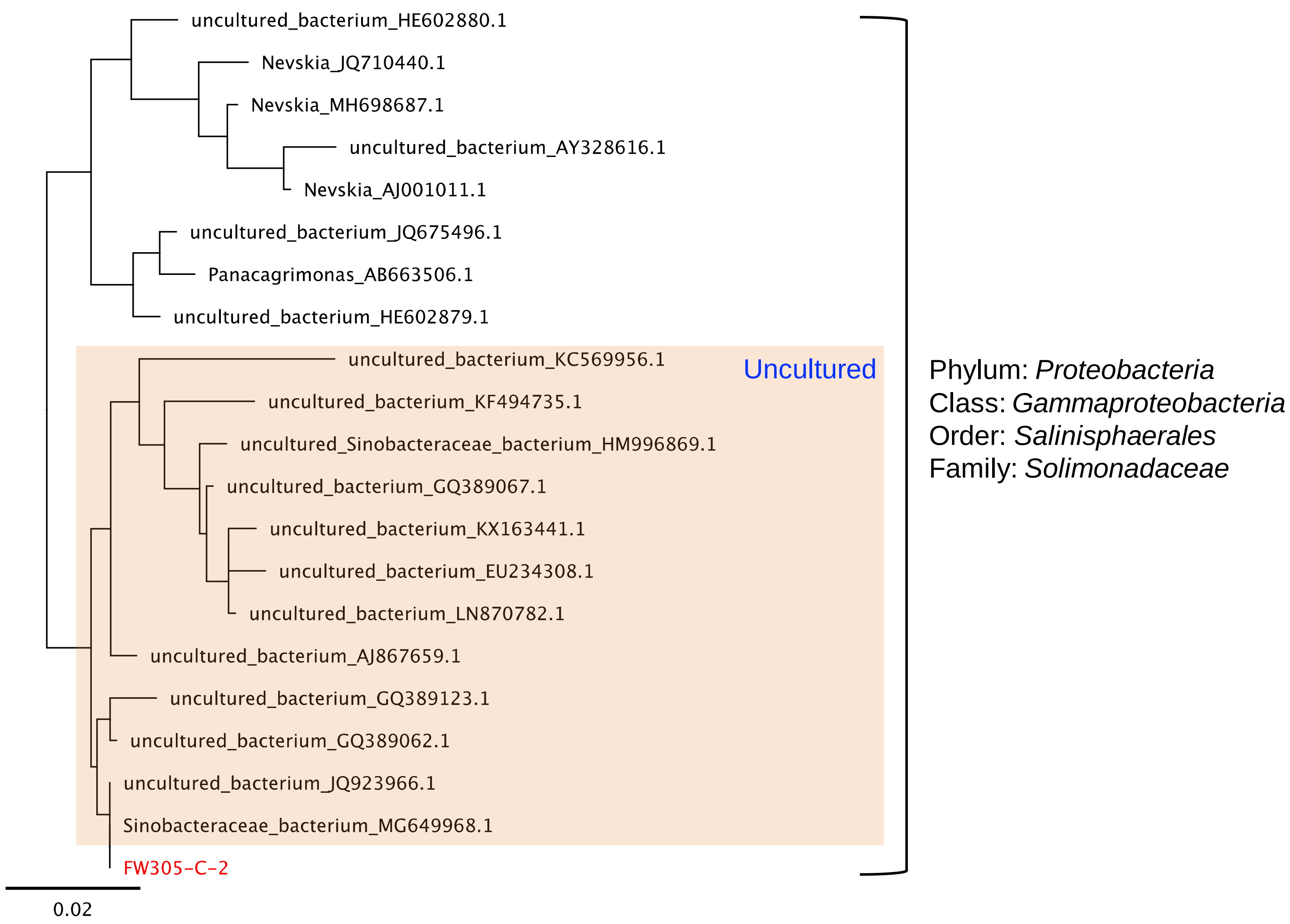
uncultured_bacterium_HE602880.1
Nevskia_JQ710440.1
Nevskia_MH698687.1
uncultured_bacterium_AY328616.1
Nevskia_AJ001011.1
uncultured_bacterium_JQ675496.1
Panacagrimonas_AB663506.1
uncultured_bacterium_HE602879.1
uncultured_bacterium_KC569956.1
Uncultured
uncultured_bacterium_KF494735.1
uncultured_Sinobacteraceae_bacterium_HM996869.1
uncultured_bacterium_GQ389067.1
uncultured_bacterium_KX163441.1
uncultured_bacterium_EU234308.1
uncultured_bacterium_LN870782.1
uncultured_bacterium_AJ867659.1
uncultured_bacterium_GQ389123.1
uncultured_bacterium_GQ389062.1
uncultured_bacterium_JQ923966.1
Sinobacteraceae_bacterium_MG649968.1
FW305-C-2
0.02
Phylum: Proteobacteria
Class: Gammaproteobacteria
Order: Salinisphaerales
Family: Solimonadaceae

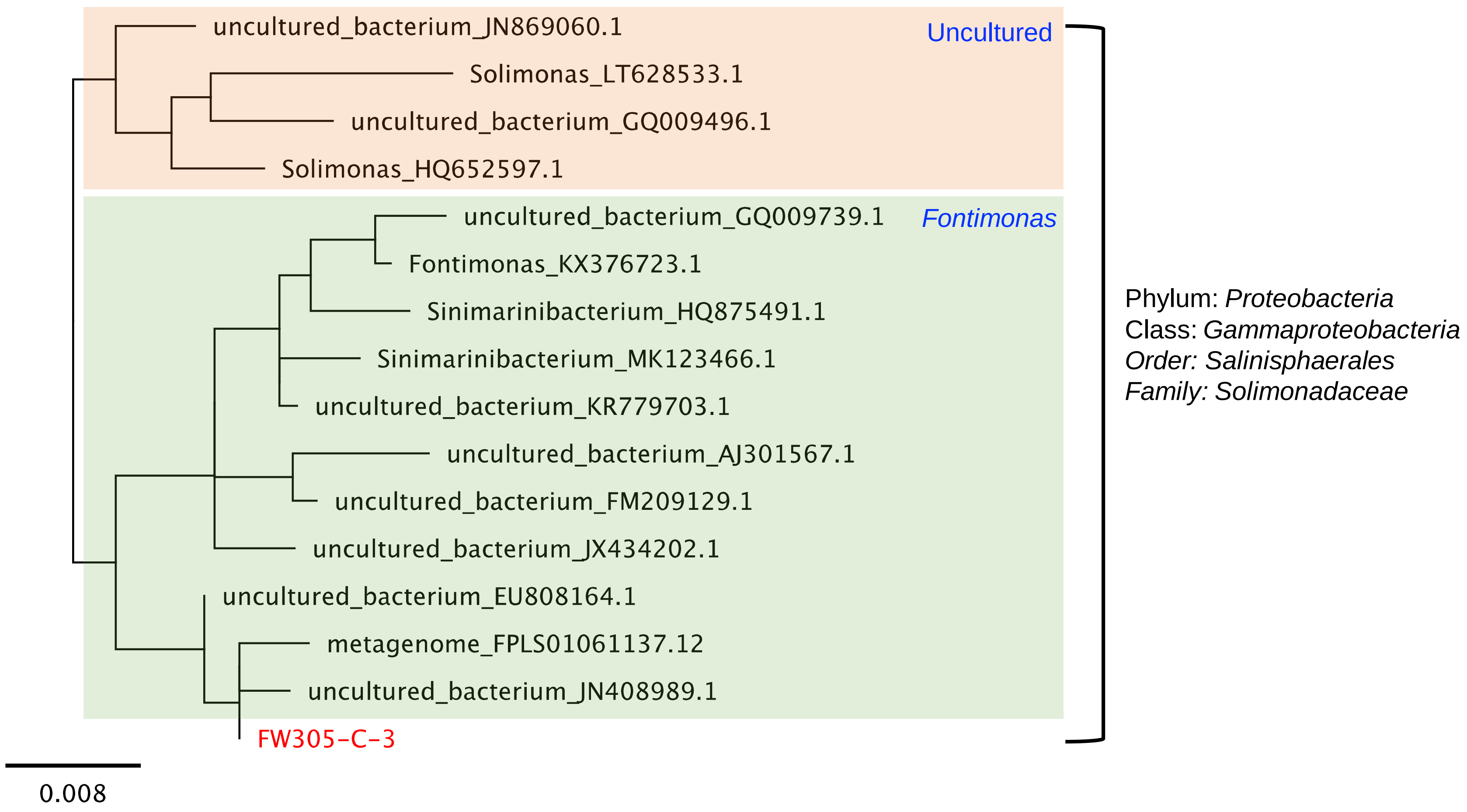

uncultured_bacterium_JN869060.1
Solimonas_LT628533.1
uncultured_bacterium_GQ009496.1
Solimonas_HQ652597.1
Uncultured
uncultured_bacterium_GQ009739.1
Fontimonas_KX376723.1
Sinimarinibacterium_HQ875491.1
Sinimarinibacterium_MK123466.1
uncultured_bacterium_KR779703.1
uncultured_bacterium_AJ301567.1
uncultured_bacterium_FM209129.1
uncultured_bacterium_JX434202.1
uncultured_bacterium_EU808164.1
metagenome_FPLS01061137.12
uncultured_bacterium_JN408989.1
FW305-C-3
Fontimonas
0.008
Phylum: Proteobacteria
Class: Gammaproteobacteria
Order: Salinisphaerales
Family: Solimonadaceae

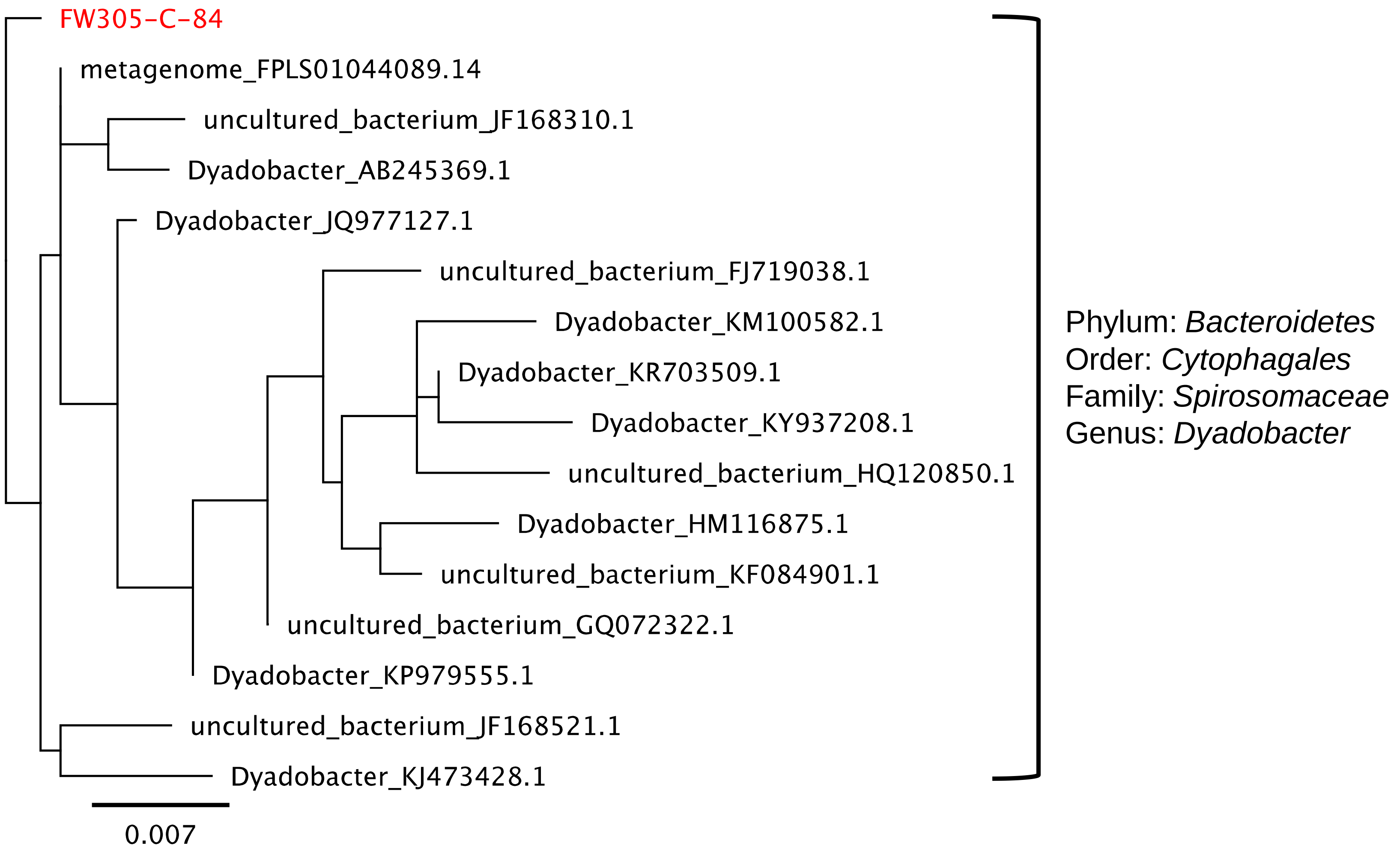
FW305-C-84
metagenome_FPLS01044089.14
uncultured_bacterium_JF168310.1
Dyadobacter_AB245369.1
Dyadobacter_JQ977127.1
uncultured_bacterium_FJ719038.1
Dyadobacter_KM100582.1
Dyadobacter_KR703509.1
Dyadobacter_KY937208.1
uncultured_bacterium_HQ120850.1
Dyadobacter_HM116875.1
uncultured_bacterium_KF084901.1
uncultured_bacterium_GQ072322.1
Dyadobacter_KP979555.1
uncultured_bacterium_JF168521.1
Dyadobacter_KJ473428.1
0.007
Phylum: *Bacteroidetes*
Order: *Cytophagales*
Family: *Spirosomaceae*
Genus: *Dyadobacter*

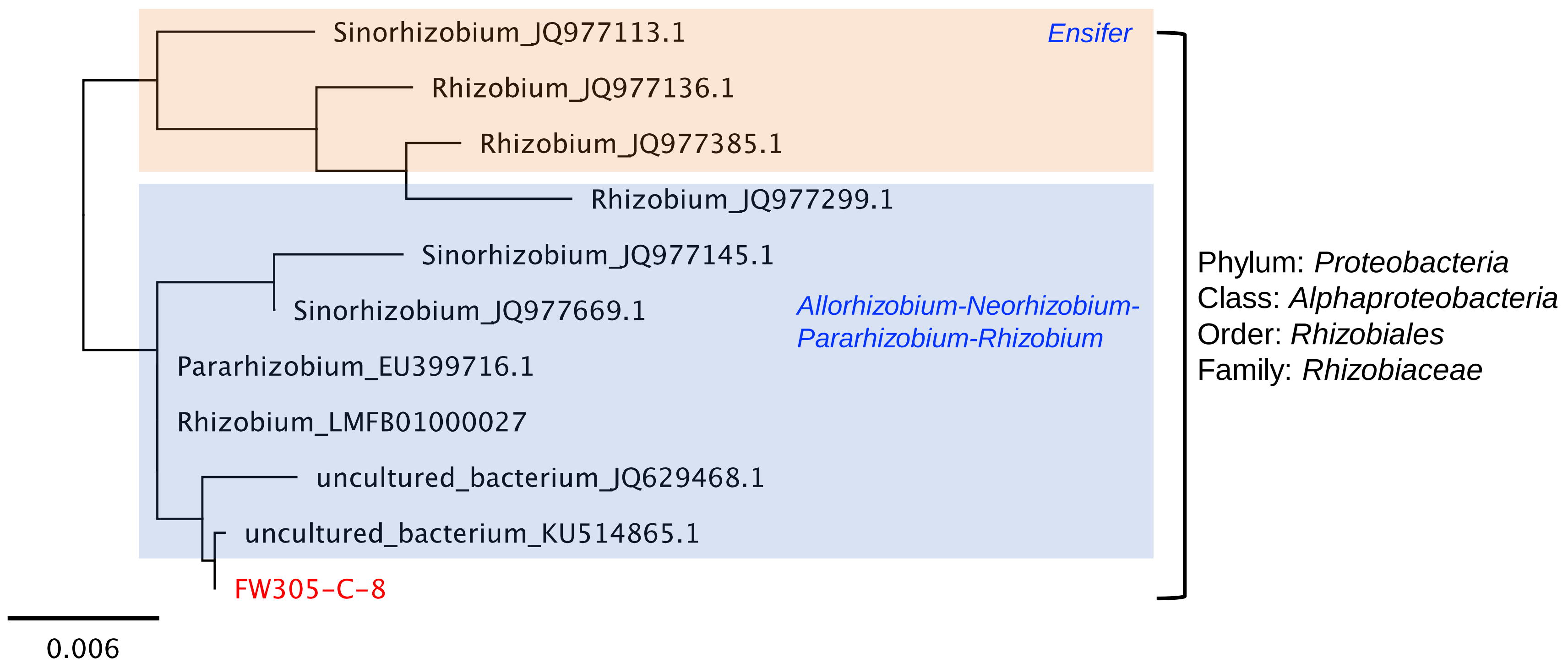
Sinorhizobium_JQ977113.1
Ensifer
Rhizobium_JQ977136.1
Rhizobium_JQ977385.1
Rhizobium_JQ977299.1
Sinorhizobium_JQ977145.1
Sinorhizobium_JQ977669.1
Allorhizobium-Neorhizobium-Pararhizobium-Rhizobium
Pararhizobium_EU399716.1
Rhizobium_LMFB01000027
uncultured_bacterium_JQ629468.1
uncultured_bacterium_KU514865.1
FW305-C-8
0.006
Phylum: Proteobacteria
Class: Alphaproteobacteria
Order: Rhizobiales
Family: Rhizobiaceae

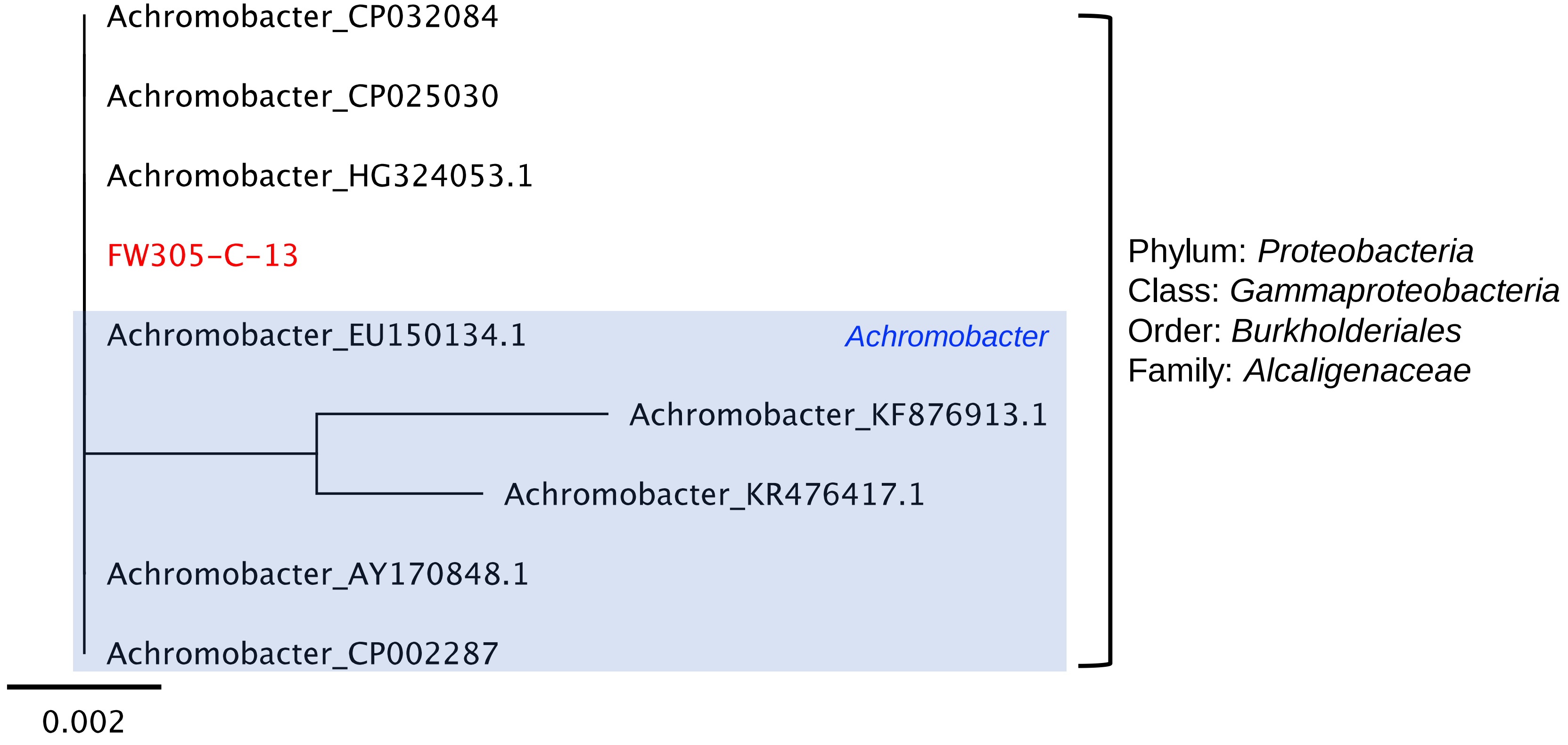

Achromobacter_CP032084
Achromobacter_CP025030
Achromobacter_HG324053.1
FW305-C-13
Achromobacter_EU150134.1
Achromobacter
Achromobacter_KF876913.1
Achromobacter_KR476417.1
Achromobacter_AY170848.1
Achromobacter_CP002287
0.002
Phylum: Proteobacteria
Class: Gammaproteobacteria
Order: Burkholderiales
Family: Alcaligenaceae

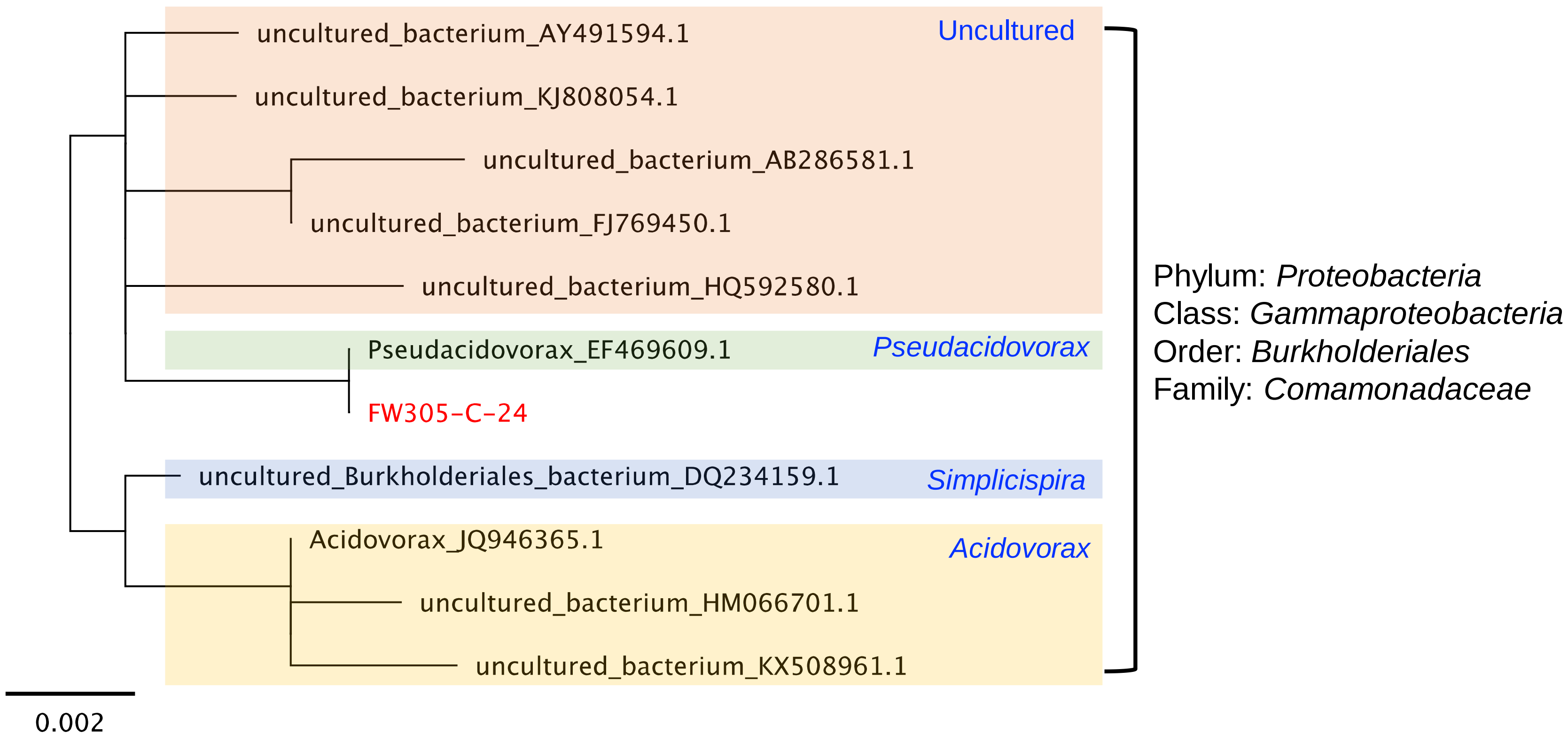

uncultured_bacterium_AY491594.1
uncultured_bacterium_KJ808054.1
uncultured_bacterium_AB286581.1
uncultured_bacterium_FJ769450.1
uncultured_bacterium_HQ592580.1
Uncultured
Pseudacidovorax_EF469609.1
Pseudacidovorax
FW305-C-24
uncultured_Burkholderiales_bacterium_DQ234159.1
Simplicispira
Acidovorax_JQ946365.1
Acidovorax
uncultured_bacterium_HM066701.1
uncultured_bacterium_KX508961.1
0.002
Phylum: Proteobacteria
Class: Gammaproteobacteria
Order: Burkholderiales
Family: Comamonadaceae

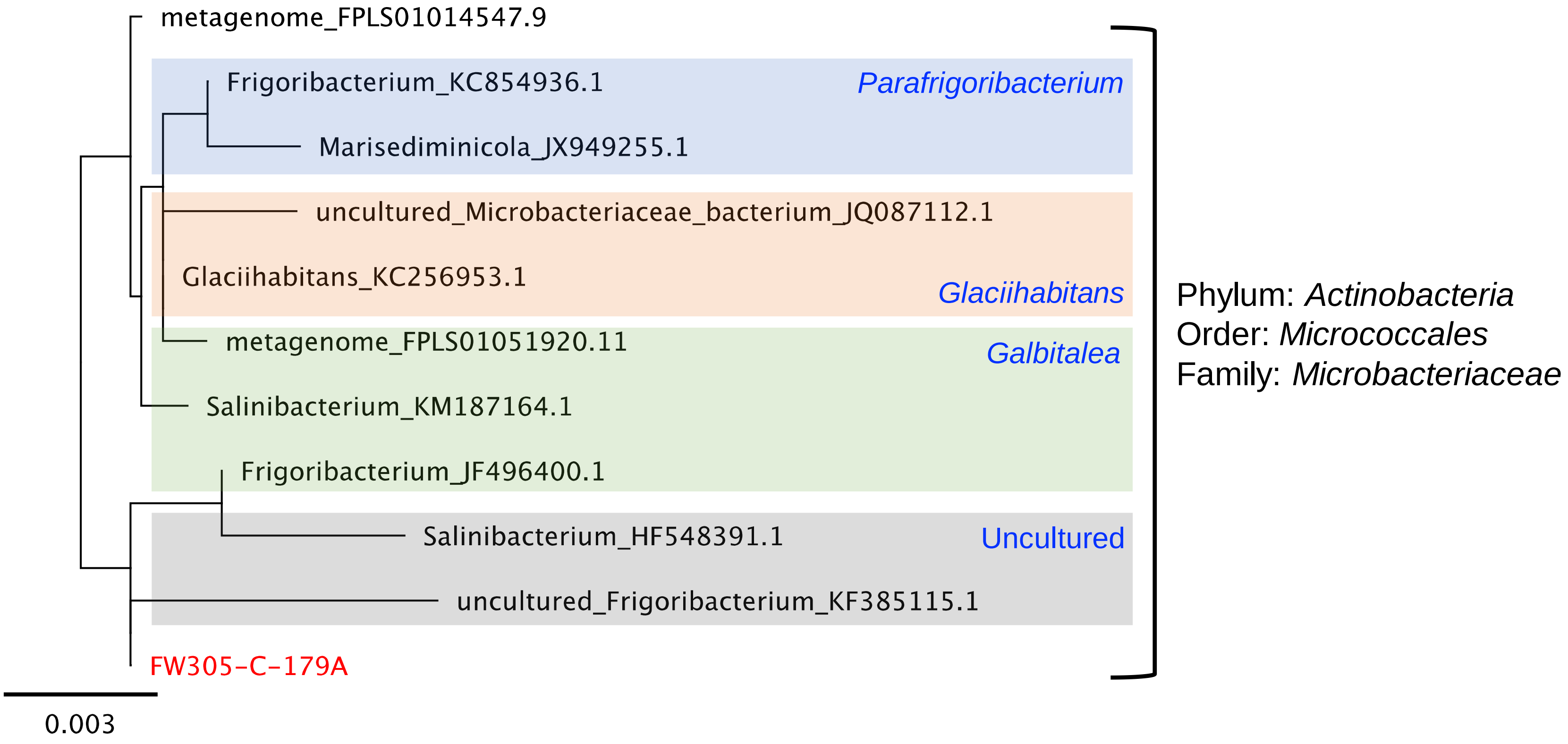
metagenome_FPLS01014547.9
Frigoribacterium_KC854936.1
Parafrigoribacterium
Marisediminicola_JX949255.1
uncultured_Microbacteriaceae_bacterium_JQ087112.1
Glaciihabitans_KC256953.1
Glaciihabitans
metagenome_FPLS01051920.11
Galbitalea
Salinibacterium_KM187164.1
Frigoribacterium_JF496400.1
Salinibacterium_HF548391.1
Uncultured
uncultured_Frigoribacterium_KF385115.1
FW305-C-179A
0.003
Phylum: Actinobacteria
Order: Micrococcales
Family: Microbacteriaceae

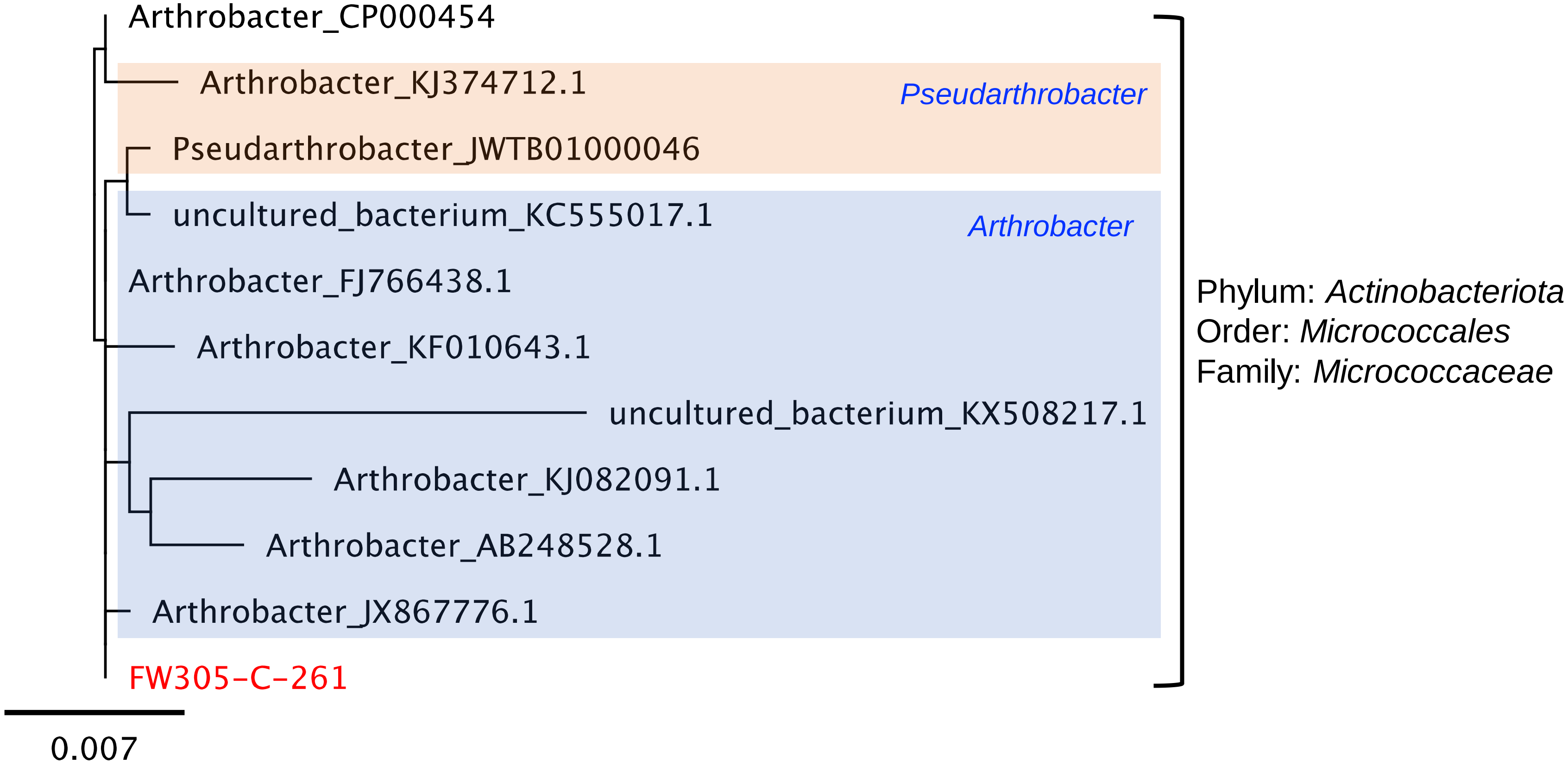
Arthrobacter_CP000454
Arthrobacter_KJ374712.1
Pseudarthrobacter
Pseudarthrobacter_JWTB01000046
uncultured_bacterium_KC555017.1
Arthrobacter
Arthrobacter_FJ766438.1
Arthrobacter_KF010643.1
uncultured_bacterium_KX508217.1
Arthrobacter_KJ082091.1
Arthrobacter_AB248528.1
Arthrobacter_JX867776.1
FW305-C-261
0.007
Phylum: *Actinobacteriota*
Order: *Micrococcales*
Family: *Micrococcaceae*

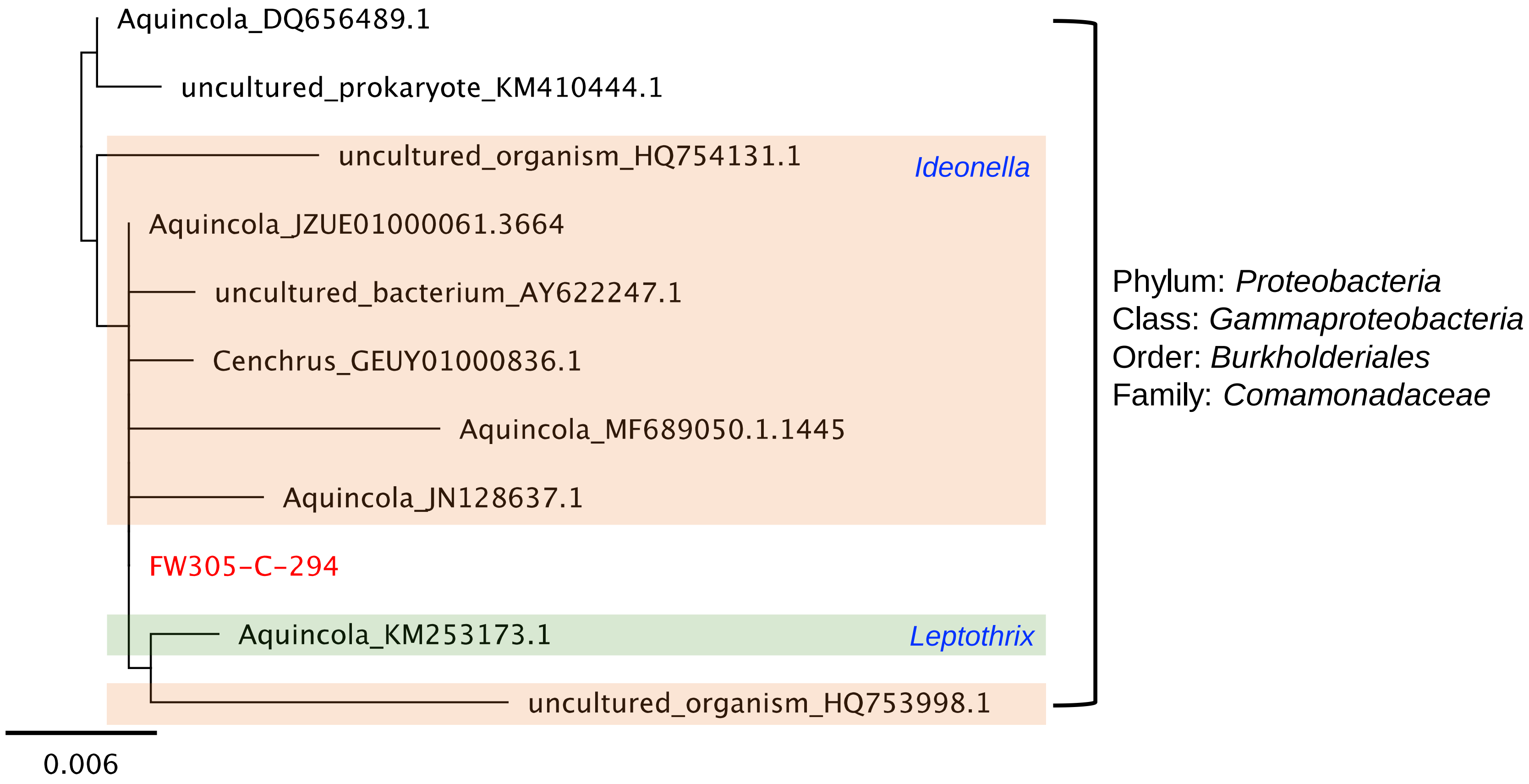

Aquincola_DQ656489.1
uncultured_prokaryote_KM410444.1
uncultured_organism_HQ754131.1
Ideonella
Aquincola_JZUE01000061.3664
uncultured_bacterium_AY622247.1
Cenchrus_GEUY01000836.1
Aquincola_MF689050.1.1445
Aquincola_JN128637.1
FW305-C-294
Aquincola_KM253173.1
Leptothrix
uncultured_organism_HQ753998.1
0.006
Phylum: *Proteobacteria*
Class: *Gammaproteobacteria*
Order: *Burkholderiales*
Family: *Comamonadaceae*

Table S1. Ingredients of vitamin stock solution.

Chemical	Concentration (mg/L)
Vitamin B_1 (Thiamine HCl)	500
Vitamin B_2 (Riboflavin)	500
Vitamin B_3 (Nicotinic acid)	500
Vitamin B_5 (D-Pantothenic acid hemicalcium)	500
Vitamin B_6 (Pyridoxine HCl)	1000
Vitamin B_7 (Biotin)	200
Vitamin B_9 (Folic acid)	200
Vitamin B_{10} (p-Amino benzoic acid)	500
Vitamin B_{12} (Cobalamin)	10
D,L-6,8-thioctic acid	500
**Total**	**4410**

Table S2. Concentration of C substrate in stock solutions and microcosms.

C substrate	Conc. in stock solution	Final conc. of amended organic C in the microcosm (mg OC/L)	Note
Glucose	200 mM (36.0 g/L)	144.1	Nominal
Sodium acetate	200 mM (16.4 g/L)	48.0	Nominal
Sodium benzoate	50 mM (7.2 g/L)	42.0	Nominal
Oleic acid	50 g/L (insoluble)	382.7 (insoluble)	Nominal
Cellulose	20 g/L (insoluble)	88.9 (insoluble)	Nominal
Mixed vitamins	4.4 g/L	24.7	Nominal
Bacterial cell lysate	TOC 2.67 g/L	26.7	Measured
Sediment DOM		48.4	Measured

Table S3. Primers used for 16S rRNA gene amplicon sequencing.

**Primer name**	**Primer sequence (5' -> 3')**
PE16S_V4_U515_F*	ACACGACGCTCTTCCGATCTYRYRGTGCCAGCMGCCGCGGTAA
PE16S_V4_E786R*	CGGCATTCCTGCTGAACCGCTCTTCCGATCTGGACTACHVGGGTWTCTAAT
PE-III-PCR-F-###	AATGATACGGCGACCACCGAGATCTACACNNNNNNNNACACTCTTTCCCTACACGACGCTCTTCCGATCT
PE-IV-PCR-R-###	CAAGCAGAAGACGGCATACGAGATNNNNNNNNCGGTCTCGGCATTCCTGCTGAACCGCTCTTCCGATCT

*Universal primer segments U515 and E786R were drawn from (Lane, 1991).

Lane, D.J. (1991) 16S/23S rRNA Sequencing. In: Stackebrandt, E. and Goodfellow, M., Eds., Nucleic Acid Techniques in Bacterial Systematic, John Wiley and Sons, New York, 115-175.

Table S4. The list of all 228 pure bacterial isolates obtained in this study.

Isolate ID	Accession number	Identity	Phylum	Class	Order	Family	Genus	C source of enrichment
FW305-C-125	MK402928	99.1	Actinobacteria	Actinobacteria	Micrococcales	Microbacteriaceae	Herbiconiux	Sed DOM
FW305-C-176A	MK402930	99.4	Actinobacteria	Actinobacteria	Micrococcales	Microbacteriaceae	Leucobacter	Cell lysate
FW305-C-176B	MN067588	99.4	Actinobacteria	Actinobacteria	Micrococcales	Microbacteriaceae	Leucobacter	Cell lysate
FW305-C-20	MK402931	99.9	Actinobacteria	Actinobacteria	Micrococcales	Microbacteriaceae	Microbacterium	Cell lysate
FW305-C-20-19	MT160389	99.2	Actinobacteria	Actinobacteria	Micrococcales	Microbacteriaceae	Microbacterium	Sed DOM
FW305-C-30-5	MT160390	99.9	Actinobacteria	Actinobacteria	Micrococcales	Microbacteriaceae	Microbacterium	Sed DOM
FW305-C-179A	MN067593	98.8	Actinobacteria	Actinobacteria	Micrococcales	Microbacteriaceae		Cell lysate
FW305-C-184B	MN067595	98.8	Actinobacteria	Actinobacteria	Micrococcales	Microbacteriaceae		Cell lysate
FW305-C-184C	MK402929	98.8	Actinobacteria	Actinobacteria	Micrococcales	Microbacteriaceae		Cell lysate
FW305-C-186A	MN067596	98.8	Actinobacteria	Actinobacteria	Micrococcales	Microbacteriaceae		Cell lysate
FW305-C-240C	MN067599	98.8	Actinobacteria	Actinobacteria	Micrococcales	Microbacteriaceae		Cell lysate
FW305-C-243A	MN067601	98.8	Actinobacteria	Actinobacteria	Micrococcales	Microbacteriaceae		Cell lysate
FW305-C-20-9	MT210153	99.8	Actinobacteria	Actinobacteria	Micrococcales	Micrococcaceae	Micrococcus	Sed DOM
FW305-C-261	MK559506	99.8	Actinobacteria	Actinobacteria	Micrococcales	Micrococcaceae		Cell lysate
FW305-C-256	MK559505	100.0	Actinobacteria	Actinobacteria	Micrococcales	Micrococcaceae	Paenarthrobacter	Sed DOM
FW305-C-257	MN067573	100.0	Actinobacteria	Actinobacteria	Micrococcales	Micrococcaceae	Paenarthrobacter	Sed DOM
FW305-C-49	MK402932	98.9	Bacteroidetes	Bacteroidia	Chitinophagales	Chitinophagaceae	Sediminibacterium	Cell lysate
FW305-C-61	MN067618	98.6	Bacteroidetes	Bacteroidia	Chitinophagales	Chitinophagaceae	Sediminibacterium	Cell lysate
FW305-C-178	MK402933	98.7	Bacteroidetes	Bacteroidia	Chitinophagales	Chitinophagaceae	Sediminibacterium	Cell lysate
FW305-C-188	MN067665	98.7	Bacteroidetes	Bacteroidia	Chitinophagales	Chitinophagaceae	Sediminibacterium	Cell lysate
FW305-C-203	MN067671	98.7	Bacteroidetes	Bacteroidia	Chitinophagales	Chitinophagaceae	Sediminibacterium	Cell lysate
FW305-C-269	MK559507	96.1	Bacteroidetes	Bacteroidia	Chitinophagales	Chitinophagaceae	Sediminibacterium	Cell lysate
FW305-C-271	MN067555	96.3	Bacteroidetes	Bacteroidia	Chitinophagales	Chitinophagaceae	Sediminibacterium	Cell lysate
FW305-C-272	MN067578	96.3	Bacteroidetes	Bacteroidia	Chitinophagales	Chitinophagaceae	Sediminibacterium	Cell lysate
FW305-C-286	MN067568	96.1	Bacteroidetes	Bacteroidia	Chitinophagales	Chitinophagaceae	Sediminibacterium	Cell lysate
FW305-C-185	MK402934	97.9	Bacteroidetes	Bacteroidia	Chitinophagales	Chitinophagaceae	Terrimonas	Cell lysate
FW305-C-84	MK402937	94.1	Bacteroidetes	Bacteroidia	Cytophagales	Spirosomaceae	Dyadobacter	Sed DOM
FW305-C-87	MN067631	94.2	Bacteroidetes	Bacteroidia	Cytophagales	Spirosomaceae	Dyadobacter	Sed DOM
FW305-C-96	MN067636	94.2	Bacteroidetes	Bacteroidia	Cytophagales	Spirosomaceae	Dyadobacter	Sed DOM
FW305-C-105	MN067638	94.2	Bacteroidetes	Bacteroidia	Cytophagales	Spirosomaceae	Dyadobacter	Sed DOM
FW305-C-110	MN067642	94.2	Bacteroidetes	Bacteroidia	Cytophagales	Spirosomaceae	Dyadobacter	Sed DOM
FW305-C-113	MN067643	94.2	Bacteroidetes	Bacteroidia	Cytophagales	Spirosomaceae	Dyadobacter	Sed DOM
FW305-C-127	MN067650	94.1	Bacteroidetes	Bacteroidia	Cytophagales	Spirosomaceae	Dyadobacter	Sed DOM
FW305-C-80	MK402936	97.9	Bacteroidetes	Bacteroidia	Cytophagales	Spirosomaceae	Emticicia	Sed DOM
FW305-C-30-33	MT160393	97.8	Bacteroidetes	Bacteroidia	Cytophagales	Spirosomaceae	Emticicia	Sed DOM
FW305-C-70	MK402935	99.0	Bacteroidetes	Bacteroidia	Cytophagales	Spirosomaceae	Flectobacillus	Sed DOM
FW305-C-79	MN067628	99.6	Bacteroidetes	Bacteroidia	Cytophagales	Spirosomaceae	Flectobacillus	Sed DOM
FW305-C-258	MN067574	99.6	Bacteroidetes	Bacteroidia	Cytophagales	Spirosomaceae	Flectobacillus	Sed DOM
FW305-C-263	MN067553	99.6	Bacteroidetes	Bacteroidia	Cytophagales	Spirosomaceae	Flectobacillus	Sed DOM
FW305-C-264	MN067554	99.6	Bacteroidetes	Bacteroidia	Cytophagales	Spirosomaceae	Flectobacillus	Sed DOM
FW305-C-265	MN067576	99.6	Bacteroidetes	Bacteroidia	Cytophagales	Spirosomaceae	Flectobacillus	Sed DOM
FW305-C-293	MN067582	99.6	Bacteroidetes	Bacteroidia	Cytophagales	Spirosomaceae	Flectobacillus	Sed DOM
FW305-C-20-21	MT160392	98.1	Bacteroidetes	Bacteroidia	Cytophagales	Spirosomaceae	Spirosoma	Sed DOM
FW305-C-10-5	MT160391	96.5	Bacteroidetes	Bacteroidia	Flavobacteriales	Flavobacteriaceae	Flavobacterium	Sed DOM
FW305-C-21	MK402939	96.3	Bacteroidetes	Bacteroidia	Sphingobacteriales	env.OPS 17		Cell lysate
FW305-C-23	MN067550	96.5	Bacteroidetes	Bacteroidia	Sphingobacteriales	env.OPS 17		Cell lysate

FW305-C-75	MK402938	98.3	Bacteroidetes	Bacteroidia	Sphingobacteriales	Sphingobacteriaceae	Pedobacter	Sed DOM
FW305-C-78	MN067627	97.9	Bacteroidetes	Bacteroidia	Sphingobacteriales	Sphingobacteriaceae	Pedobacter	Sed DOM
FW305-C-277	MN067579	97.7	Bacteroidetes	Bacteroidia	Sphingobacteriales	Sphingobacteriaceae	Pedobacter	Sed DOM
FW305-C-10-2	MT160394	100.0	Deinococcota	Deinococci	Deinococcales	Deinococcaceae	Deinococcus	Sed DOM
FW305-C-20-7	MT160395	100.0	Deinococcota	Deinococci	Deinococcales	Deinococcaceae	Deinococcus	Sed DOM
FW305-C-191	MK402940	99.6	Firmicutes	Bacilli	Bacillales	Bacillaceae	Bacillus	Cell lysate
FW305-C-196	MN067667	99.6	Firmicutes	Bacilli	Bacillales	Bacillaceae	Bacillus	Sed DOM
FW305-C-190	MK402943	99.3	Firmicutes	Bacilli	Bacillales	Paenibacillaceae	Paenibacillus	Cell lysate
FW305-C-202	MK402944	99.7	Firmicutes	Bacilli	Bacillales	Paenibacillaceae	Paenibacillus	Cell lysate
FW305-C-1	MK402942	100.0	Firmicutes	Bacilli	Brevibacillales	Brevibacillaceae	Brevibacillus	Cell lysate
FW305-C-11	MN067603	99.9	Firmicutes	Bacilli	Brevibacillales	Brevibacillaceae	Brevibacillus	Cell lysate
FW305-C-34	MN067608	100.0	Firmicutes	Bacilli	Brevibacillales	Brevibacillaceae	Brevibacillus	Cell lysate
FW305-C-48	MK402941	99.7	Firmicutes	Bacilli	Brevibacillales	Brevibacillaceae	Brevibacillus	Cell lysate
FW305-C-62	MN067619	100.0	Firmicutes	Bacilli	Brevibacillales	Brevibacillaceae	Brevibacillus	Cell lysate
FW305-C-68	MN067622	99.9	Firmicutes	Bacilli	Brevibacillales	Brevibacillaceae	Brevibacillus	Sed DOM
FW305-C-69	MN067623	100.0	Firmicutes	Bacilli	Brevibacillales	Brevibacillaceae	Brevibacillus	Sed DOM
FW305-C-72	MN067624	100.0	Firmicutes	Bacilli	Brevibacillales	Brevibacillaceae	Brevibacillus	Sed DOM
FW305-C-126	MN067649	100.0	Firmicutes	Bacilli	Brevibacillales	Brevibacillaceae	Brevibacillus	Sed DOM
FW305-C-192	MN067666	99.7	Firmicutes	Bacilli	Brevibacillales	Brevibacillaceae	Brevibacillus	Cell lysate
FW305-C-197	MN067668	99.9	Firmicutes	Bacilli	Brevibacillales	Brevibacillaceae	Brevibacillus	Sed DOM
FW305-C-200	MN067669	100.0	Firmicutes	Bacilli	Brevibacillales	Brevibacillaceae	Brevibacillus	Cell lysate
FW305-C-201	MN067670	100.0	Firmicutes	Bacilli	Brevibacillales	Brevibacillaceae	Brevibacillus	Cell lysate
FW305-C-205	MN067672	100.0	Firmicutes	Bacilli	Brevibacillales	Brevibacillaceae	Brevibacillus	Sed DOM
FW305-C-206	MN067673	100.0	Firmicutes	Bacilli	Brevibacillales	Brevibacillaceae	Brevibacillus	Sed DOM
FW305-C-207	MN067674	100.0	Firmicutes	Bacilli	Brevibacillales	Brevibacillaceae	Brevibacillus	Sed DOM
FW305-C-208	MN067675	100.0	Firmicutes	Bacilli	Brevibacillales	Brevibacillaceae	Brevibacillus	Sed DOM
FW305-C-209	MN067676	100.0	Firmicutes	Bacilli	Brevibacillales	Brevibacillaceae	Brevibacillus	Sed DOM
FW305-C-210	MN067677	100.0	Firmicutes	Bacilli	Brevibacillales	Brevibacillaceae	Brevibacillus	Sed DOM
FW305-C-211	MN067678	99.9	Firmicutes	Bacilli	Brevibacillales	Brevibacillaceae	Brevibacillus	Sed DOM
FW305-C-103	MK402958	98.0	Proteobacteria	Alphaproteobacteria	Acetobacterales	Acetobacteraceae	Roseomonas	Sed DOM
FW305-C-119	MK402959	97.5	Proteobacteria	Alphaproteobacteria	Acetobacterales	Acetobacteraceae	Roseomonas	Sed DOM
FW305-C-136D	MN305721	95.8	Proteobacteria	Alphaproteobacteria	Acetobacterales	Acetobacteraceae	Roseococcus	Sed DOM
FW305-C-18	MK402945	100.0	Proteobacteria	Alphaproteobacteria	Caulobacterales	Caulobacteraceae	Brevundimonas	Cell lysate
FW305-C-20-1	MT160402	100.0	Proteobacteria	Alphaproteobacteria	Caulobacterales	Caulobacteraceae	Brevundimonas	Sed DOM
FW305-C-30-13	MT160403	99.9	Proteobacteria	Alphaproteobacteria	Caulobacterales	Caulobacteraceae	Brevundimonas	Sed DOM
FW305-C-128	MK402946	99.5	Proteobacteria	Alphaproteobacteria	Caulobacterales	Caulobacteraceae	Caulobacter	Sed DOM
FW305-C-130	MK402947	98.3	Proteobacteria	Alphaproteobacteria	Caulobacterales	Caulobacteraceae	Caulobacter	Sed DOM
FW305-C-30-S1	MT538329	98.1	Proteobacteria	Alphaproteobacteria	Caulobacterales	Caulobacteraceae	Phenylobacterium	Sed DOM
FW305-C-30-S3	MT538330	98.9	Proteobacteria	Alphaproteobacteria	Reyranellales	Reyranellaceae	Reyranella	Sed DOM
FW305-C-30-S5	MT538331	98.9	Proteobacteria	Alphaproteobacteria	Reyranellales	Reyranellaceae	Reyranella	Sed DOM
FW305-C-30-S8	MT538332	98.9	Proteobacteria	Alphaproteobacteria	Reyranellales	Reyranellaceae	Reyranella	Sed DOM
FW305-C-30-S9	MT538333	98.9	Proteobacteria	Alphaproteobacteria	Reyranellales	Reyranellaceae	Reyranella	Sed DOM
FW305-C-30-S10	MT538334	98.9	Proteobacteria	Alphaproteobacteria	Reyranellales	Reyranellaceae	Reyranella	Sed DOM
FW305-C-74	MK402952	100.0	Proteobacteria	Alphaproteobacteria	Rhizobiales	Beijerinckiaceae	Bosea	Sed DOM
FW305-C-92	MK402950	99.9	Proteobacteria	Alphaproteobacteria	Rhizobiales	Beijerinckiaceae	Bosea	Sed DOM
FW305-C-101	MK402951	99.7	Proteobacteria	Alphaproteobacteria	Rhizobiales	Beijerinckiaceae	Bosea	Sed DOM
FW305-C-116	MN067645	99.9	Proteobacteria	Alphaproteobacteria	Rhizobiales	Beijerinckiaceae	Bosea	Sed DOM
FW305-C-117	MN067646	100.0	Proteobacteria	Alphaproteobacteria	Rhizobiales	Beijerinckiaceae	Bosea	Sed DOM

FW305-C-121	MN067647	100.0	Proteobacteria	Alphaproteobacteria	Rhizobiales	Beijerinckiaceae	Bosea	Sed DOM
FW305-C-275	MN067558	100.0	Proteobacteria	Alphaproteobacteria	Rhizobiales	Beijerinckiaceae	Bosea	Sed DOM
FW305-C-198	MK402954	99.7	Proteobacteria	Alphaproteobacteria	Rhizobiales	Beijerinckiaceae	Methylobacterium-Methylorubrum	Sed DOM
FW305-C-47	MK402953	99.2	Proteobacteria	Alphaproteobacteria	Rhizobiales	Devosiaceae	Devosia	Cell lysate
FW305-C-77	MN067626	99.1	Proteobacteria	Alphaproteobacteria	Rhizobiales	Devosiaceae	Devosia	Sed DOM
FW305-C-91	MN067634	99.1	Proteobacteria	Alphaproteobacteria	Rhizobiales	Devosiaceae	Devosia	Sed DOM
FW305-C-93	MN067635	99.1	Proteobacteria	Alphaproteobacteria	Rhizobiales	Devosiaceae	Devosia	Sed DOM
FW305-C-99	MN067637	99.1	Proteobacteria	Alphaproteobacteria	Rhizobiales	Devosiaceae	Devosia	Sed DOM
FW305-C-106	MN067639	99.1	Proteobacteria	Alphaproteobacteria	Rhizobiales	Devosiaceae	Devosia	Sed DOM
FW305-C-123	MN067648	99.1	Proteobacteria	Alphaproteobacteria	Rhizobiales	Devosiaceae	Devosia	Sed DOM
FW305-C-141	MN067653	99.4	Proteobacteria	Alphaproteobacteria	Rhizobiales	Devosiaceae	Devosia	Sed DOM
FW305-C-149	MN067659	98.8	Proteobacteria	Alphaproteobacteria	Rhizobiales	Devosiaceae	Devosia	Sed DOM
FW305-C-156	MN067663	99.3	Proteobacteria	Alphaproteobacteria	Rhizobiales	Devosiaceae	Devosia	Sed DOM
FW305-C-176I	MK402957	99.5	Proteobacteria	Alphaproteobacteria	Rhizobiales	Rhizobiaceae	Allorhizobium-Neorhizobium-Pararhizobium-Rhizobium	Cell lysate
FW305-C-52	MK402949	100.0	Proteobacteria	Alphaproteobacteria	Rhizobiales	Rhizobiaceae	Aminobacter	Cell lysate
FW305-C-20-5	MT160400	99.9	Proteobacteria	Alphaproteobacteria	Rhizobiales	Rhizobiaceae	Aminobacter	Sed DOM
FW305-C-10-16	MT160396	99.9	Proteobacteria	Alphaproteobacteria	Rhizobiales	Rhizobiaceae	Ensifer	Sed DOM
FW305-C-10-9	MT160397	99.7	Proteobacteria	Alphaproteobacteria	Rhizobiales	Rhizobiaceae	Ensifer	Sed DOM
FW305-C-20-14	MT160398	98.9	Proteobacteria	Alphaproteobacteria	Rhizobiales	Rhizobiaceae	Shinella	Sed DOM
FW305-C-30-9	MT160399	98.9	Proteobacteria	Alphaproteobacteria	Rhizobiales	Rhizobiaceae	Shinella	Sed DOM
FW305-C-8	MK402956	95.9	Proteobacteria	Alphaproteobacteria	Rhizobiales	Rhizobiaceae		Cell lysate
FW305-C-26	MN067583	95.9	Proteobacteria	Alphaproteobacteria	Rhizobiales	Rhizobiaceae		Cell lysate
FW305-C-27	MN067584	95.9	Proteobacteria	Alphaproteobacteria	Rhizobiales	Rhizobiaceae		Cell lysate
FW305-C-30	MN067585	95.9	Proteobacteria	Alphaproteobacteria	Rhizobiales	Rhizobiaceae		Cell lysate
FW305-C-39	MN067586	95.9	Proteobacteria	Alphaproteobacteria	Rhizobiales	Rhizobiaceae		Cell lysate
FW305-C-43	MN067587	95.9	Proteobacteria	Alphaproteobacteria	Rhizobiales	Rhizobiaceae		Cell lysate
FW305-C-134A	MK402966	98.4	Proteobacteria	Alphaproteobacteria	Rhizobiales	Rhizobiales-Incertae-Sedis	Phreatobacter	Sed DOM
FW305-C-112	MK402955	99.8	Proteobacteria	Alphaproteobacteria	Rhizobiales	Rhizobiales-Incertae-Sedis	Nordella	Sed DOM
FW305-C-122	MK402948	99.6	Proteobacteria	Alphaproteobacteria	Rhizobiales	Xanthobacteraceae	Afipia	Sed DOM
FW305-C-53	MK402962	99.9	Proteobacteria	Alphaproteobacteria	Sphingomonadales	Sphingomonadaceae	Novosphingobium	Cell lysate
FW305-C-63	MN067620	99.9	Proteobacteria	Alphaproteobacteria	Sphingomonadales	Sphingomonadaceae	Novosphingobium	Cell lysate
FW305-C-66	MN067621	99.9	Proteobacteria	Alphaproteobacteria	Sphingomonadales	Sphingomonadaceae	Novosphingobium	Cell lysate
FW305-C-71	MK402960	99.0	Proteobacteria	Alphaproteobacteria	Sphingomonadales	Sphingomonadaceae	Novosphingobium	Sed DOM
FW305-C-76	MN067625	98.6	Proteobacteria	Alphaproteobacteria	Sphingomonadales	Sphingomonadaceae	Novosphingobium	Sed DOM
FW305-C-111	MK402961	98.7	Proteobacteria	Alphaproteobacteria	Sphingomonadales	Sphingomonadaceae	Novosphingobium	Sed DOM
FW305-C-137	MN067651	99.0	Proteobacteria	Alphaproteobacteria	Sphingomonadales	Sphingomonadaceae	Novosphingobium	Sed DOM
FW305-C-184A	MN067594	99.9	Proteobacteria	Alphaproteobacteria	Sphingomonadales	Sphingomonadaceae	Novosphingobium	Cell lysate
FW305-C-240A	MN067597	99.9	Proteobacteria	Alphaproteobacteria	Sphingomonadales	Sphingomonadaceae	Novosphingobium	Cell lysate
FW305-C-247	MK559503	98.4	Proteobacteria	Alphaproteobacteria	Sphingomonadales	Sphingomonadaceae	Novosphingobium	Sed DOM
FW305-C-291	MN067570	98.4	Proteobacteria	Alphaproteobacteria	Sphingomonadales	Sphingomonadaceae	Novosphingobium	Sed DOM
FW305-C-56	MK402964	99.3	Proteobacteria	Alphaproteobacteria	Sphingomonadales	Sphingomonadaceae	Sphingomonas	Cell lysate
FW305-C-94	MK402963	99.3	Proteobacteria	Alphaproteobacteria	Sphingomonadales	Sphingomonadaceae	Sphingomonas	Sed DOM
FW305-C-54	MK402965	99.0	Proteobacteria	Alphaproteobacteria	Sphingomonadales	Sphingomonadaceae	Sphingopyxis	Cell lysate
FW305-C-20-25	MT160401	98.5	Proteobacteria	Alphaproteobacteria	Sphingomonadales	Sphingomonadaceae	Sphingopyxis	Sed DOM
FW305-C-13	MN067605	99.9	Proteobacteria	Gammaproteobacteria	Burkholderiales	Alcaligenaceae		Cell lysate
FW305-C-28	MK402967	99.9	Proteobacteria	Gammaproteobacteria	Burkholderiales	Alcaligenaceae		Cell lysate
FW305-C-31	MK402968	99.9	Proteobacteria	Gammaproteobacteria	Burkholderiales	Alcaligenaceae		Cell lysate
FW305-C-107	MN067640	99.9	Proteobacteria	Gammaproteobacteria	Burkholderiales	Alcaligenaceae		Sed DOM

FW305-C-114	MN067644	99.9	Proteobacteria	Gammaproteobacteria	Burkholderiales	Alcaligenaceae		Sed DOM
FW305-C-287	MN067580	99.9	Proteobacteria	Gammaproteobacteria	Burkholderiales	Alcaligenaceae		Cell lysate
FW305-C-176C	MK402970	99.6	Proteobacteria	Gammaproteobacteria	Burkholderiales	Burkholderiaceae	Cupriavidus	Cell lysate
FW305-C-176D	MN067589	99.6	Proteobacteria	Gammaproteobacteria	Burkholderiales	Burkholderiaceae	Cupriavidus	Cell lysate
FW305-C-176E	MN067590	99.6	Proteobacteria	Gammaproteobacteria	Burkholderiales	Burkholderiaceae	Cupriavidus	Cell lysate
FW305-C-176F	MN067591	99.6	Proteobacteria	Gammaproteobacteria	Burkholderiales	Burkholderiaceae	Cupriavidus	Cell lysate
FW305-C-176H	MN067592	99.6	Proteobacteria	Gammaproteobacteria	Burkholderiales	Burkholderiaceae	Cupriavidus	Cell lysate
FW305-C-25	MK402969	99.1	Proteobacteria	Gammaproteobacteria	Burkholderiales	Comamonadaceae	Acidovorax	Cell lysate
FW305-C-20-26	MT160404	99.6	Proteobacteria	Gammaproteobacteria	Burkholderiales	Comamonadaceae	Acidovorax	Sed DOM
FW305-C-255	MN067551	100.0	Proteobacteria	Gammaproteobacteria	Burkholderiales	Comamonadaceae	Variovorax	Cell lysate
FW305-C-260	MN067552	100.0	Proteobacteria	Gammaproteobacteria	Burkholderiales	Comamonadaceae	Variovorax	Cell lysate
FW305-C-254	MN067572	100.0	Proteobacteria	Gammaproteobacteria	Burkholderiales	Comamonadaceae	Variovorax	Cell lysate
FW305-C-262	MN067575	100.0	Proteobacteria	Gammaproteobacteria	Burkholderiales	Comamonadaceae	Variovorax	Cell lysate
FW305-C-15	MN067606	100.0	Proteobacteria	Gammaproteobacteria	Burkholderiales	Comamonadaceae	Variovorax	Cell lysate
FW305-C-7	MK402973	100.0	Proteobacteria	Gammaproteobacteria	Burkholderiales	Comamonadaceae	Variovorax	Cell lysate
FW305-C-288	MN067581	100.0	Proteobacteria	Gammaproteobacteria	Burkholderiales	Comamonadaceae	Variovorax	Cell lysate
FW305-C-136	MK402971	98.9	Proteobacteria	Gammaproteobacteria	Burkholderiales	Comamonadaceae		Sed DOM
FW305-C-140	MN067652	98.9	Proteobacteria	Gammaproteobacteria	Burkholderiales	Comamonadaceae		Sed DOM
FW305-C-142	MN067654	98.9	Proteobacteria	Gammaproteobacteria	Burkholderiales	Comamonadaceae		Sed DOM
FW305-C-143	MN067655	98.9	Proteobacteria	Gammaproteobacteria	Burkholderiales	Comamonadaceae		Sed DOM
FW305-C-144	MN067656	98.9	Proteobacteria	Gammaproteobacteria	Burkholderiales	Comamonadaceae		Sed DOM
FW305-C-145	MN067657	98.9	Proteobacteria	Gammaproteobacteria	Burkholderiales	Comamonadaceae		Sed DOM
FW305-C-146	MN067658	98.7	Proteobacteria	Gammaproteobacteria	Burkholderiales	Comamonadaceae		Sed DOM
FW305-C-152	MN067660	98.9	Proteobacteria	Gammaproteobacteria	Burkholderiales	Comamonadaceae		Sed DOM
FW305-C-154	MN067661	98.8	Proteobacteria	Gammaproteobacteria	Burkholderiales	Comamonadaceae		Sed DOM
FW305-C-155	MN067662	98.9	Proteobacteria	Gammaproteobacteria	Burkholderiales	Comamonadaceae		Sed DOM
FW305-C-159	MN067664	98.9	Proteobacteria	Gammaproteobacteria	Burkholderiales	Comamonadaceae		Sed DOM
FW305-C-213	MN067679	98.9	Proteobacteria	Gammaproteobacteria	Burkholderiales	Comamonadaceae		Sed DOM
FW305-C-214	MN067680	98.9	Proteobacteria	Gammaproteobacteria	Burkholderiales	Comamonadaceae		Sed DOM
FW305-C-215	MN067681	98.9	Proteobacteria	Gammaproteobacteria	Burkholderiales	Comamonadaceae		Sed DOM
FW305-C-218	MN067682	98.9	Proteobacteria	Gammaproteobacteria	Burkholderiales	Comamonadaceae		Sed DOM
FW305-C-219	MN067683	98.9	Proteobacteria	Gammaproteobacteria	Burkholderiales	Comamonadaceae		Sed DOM
FW305-C-221	MN067684	98.9	Proteobacteria	Gammaproteobacteria	Burkholderiales	Comamonadaceae		Sed DOM
FW305-C-222	MN067685	98.9	Proteobacteria	Gammaproteobacteria	Burkholderiales	Comamonadaceae		Sed DOM
FW305-C-225	MN067686	98.9	Proteobacteria	Gammaproteobacteria	Burkholderiales	Comamonadaceae		Sed DOM
FW305-C-226	MN067687	98.9	Proteobacteria	Gammaproteobacteria	Burkholderiales	Comamonadaceae		Sed DOM
FW305-C-227	MN067688	98.9	Proteobacteria	Gammaproteobacteria	Burkholderiales	Comamonadaceae		Sed DOM
FW305-C-229	MN067689	98.9	Proteobacteria	Gammaproteobacteria	Burkholderiales	Comamonadaceae		Sed DOM
FW305-C-240B	MN067598	98.9	Proteobacteria	Gammaproteobacteria	Burkholderiales	Comamonadaceae		Cell lysate
FW305-C-240D	MN067600	98.9	Proteobacteria	Gammaproteobacteria	Burkholderiales	Comamonadaceae		Cell lysate
FW305-C-24	MK402972	99.9	Proteobacteria	Gammaproteobacteria	Burkholderiales	Comamonadaceae		Cell lysate
FW305-C-268	MN067577	99.9	Proteobacteria	Gammaproteobacteria	Burkholderiales	Comamonadaceae		Sed DOM
FW305-C-29	MN067607	99.9	Proteobacteria	Gammaproteobacteria	Burkholderiales	Comamonadaceae		Cell lysate
FW305-C-41	MN067610	99.9	Proteobacteria	Gammaproteobacteria	Burkholderiales	Comamonadaceae		Cell lysate
FW305-C-83	MN067629	99.9	Proteobacteria	Gammaproteobacteria	Burkholderiales	Comamonadaceae		Sed DOM
FW305-C-90	MN067633	99.9	Proteobacteria	Gammaproteobacteria	Burkholderiales	Comamonadaceae		Sed DOM
FW305-C-294	MK559509	98.5	Proteobacteria	Gammaproteobacteria	Burkholderiales	Comamonadaceae		Sed DOM
FW305-C-10-12	MT160405	100.0	Proteobacteria	Gammaproteobacteria	Burkholderiales	Comamonadaceae		Sed DOM

FW305-C-10-24	MT160406	99.7	Proteobacteria	Gammaproteobacteria	Burkholderiales	Comamonadaceae		Sed DOM
FW305-C-20-3	MT160407	99.9	Proteobacteria	Gammaproteobacteria	Burkholderiales	Comamonadaceae		Sed DOM
FW305-C-30-18	MT160408	99.7	Proteobacteria	Gammaproteobacteria	Burkholderiales	Comamonadaceae		Sed DOM
FW305-C-30-23	MT160409	99.9	Proteobacteria	Gammaproteobacteria	Burkholderiales	Comamonadaceae		Sed DOM
FW305-C-19	MK402974	99.9	Proteobacteria	Gammaproteobacteria	Burkholderiales	Rhodocyclaceae	Dechloromonas	Cell lysate
FW305-C-5	MK402978	100.0	Proteobacteria	Gammaproteobacteria	Pseudomonadales	Pseudomonadaceae	Pseudomonas	Cell lysate
FW305-C-12	MN067604	100.0	Proteobacteria	Gammaproteobacteria	Pseudomonadales	Pseudomonadaceae	Pseudomonas	Cell lysate
FW305-C-40	MK402975	99.1	Proteobacteria	Gammaproteobacteria	Salinisphaerales	Solimonadaceae	Hydrocarboniphaga	Cell lysate
FW305-C-50	MN067611	99.1	Proteobacteria	Gammaproteobacteria	Salinisphaerales	Solimonadaceae	Hydrocarboniphaga	Cell lysate
FW305-C-51	MN067612	99.1	Proteobacteria	Gammaproteobacteria	Salinisphaerales	Solimonadaceae	Hydrocarboniphaga	Cell lysate
FW305-C-55	MN067613	99.1	Proteobacteria	Gammaproteobacteria	Salinisphaerales	Solimonadaceae	Hydrocarboniphaga	Cell lysate
FW305-C-57	MN067614	99.1	Proteobacteria	Gammaproteobacteria	Salinisphaerales	Solimonadaceae	Hydrocarboniphaga	Cell lysate
FW305-C-58	MN067615	99.1	Proteobacteria	Gammaproteobacteria	Salinisphaerales	Solimonadaceae	Hydrocarboniphaga	Cell lysate
FW305-C-59	MN067616	99.1	Proteobacteria	Gammaproteobacteria	Salinisphaerales	Solimonadaceae	Hydrocarboniphaga	Cell lysate
FW305-C-10	MN067602	93.3	Proteobacteria	Gammaproteobacteria	Salinisphaerales	Solimonadaceae	uncultured	Cell lysate
FW305-C-60	MN067617	93.3	Proteobacteria	Gammaproteobacteria	Salinisphaerales	Solimonadaceae	uncultured	Cell lysate
FW305-C-270	MK559508	93.5	Proteobacteria	Gammaproteobacteria	Salinisphaerales	Solimonadaceae	uncultured	Cell lysate
FW305-C-274	MN067557	93.3	Proteobacteria	Gammaproteobacteria	Salinisphaerales	Solimonadaceae	uncultured	Sed DOM
FW305-C-276	MN067559	93.3	Proteobacteria	Gammaproteobacteria	Salinisphaerales	Solimonadaceae	uncultured	Sed DOM
FW305-C-278	MN067560	93.3	Proteobacteria	Gammaproteobacteria	Salinisphaerales	Solimonadaceae	uncultured	Cell lysate
FW305-C-279	MN067561	93.3	Proteobacteria	Gammaproteobacteria	Salinisphaerales	Solimonadaceae	uncultured	Cell lysate
FW305-C-280	MN067562	93.3	Proteobacteria	Gammaproteobacteria	Salinisphaerales	Solimonadaceae	uncultured	Cell lysate
FW305-C-281	MN067563	93.5	Proteobacteria	Gammaproteobacteria	Salinisphaerales	Solimonadaceae	uncultured	Sed DOM
FW305-C-282	MN067564	93.3	Proteobacteria	Gammaproteobacteria	Salinisphaerales	Solimonadaceae	uncultured	Sed DOM
FW305-C-284	MN067566	93.4	Proteobacteria	Gammaproteobacteria	Salinisphaerales	Solimonadaceae	uncultured	Sed DOM
FW305-C-290	MN067569	93.3	Proteobacteria	Gammaproteobacteria	Salinisphaerales	Solimonadaceae	uncultured	Sed DOM
FW305-C-292	MN067571	93.5	Proteobacteria	Gammaproteobacteria	Salinisphaerales	Solimonadaceae	uncultured	Sed DOM
FW305-C-2	MK402976	93.3	Proteobacteria	Gammaproteobacteria	Salinisphaerales	Solimonadaceae		Cell lysate
FW305-C-35	MN067609	93.3	Proteobacteria	Gammaproteobacteria	Salinisphaerales	Solimonadaceae		Cell lysate
FW305-C-86	MN067630	93.5	Proteobacteria	Gammaproteobacteria	Salinisphaerales	Solimonadaceae		Sed DOM
FW305-C-89	MN067632	93.3	Proteobacteria	Gammaproteobacteria	Salinisphaerales	Solimonadaceae		Sed DOM
FW305-C-108	MN067641	93.3	Proteobacteria	Gammaproteobacteria	Salinisphaerales	Solimonadaceae		Sed DOM
FW305-C-273	MN067556	93.3	Proteobacteria	Gammaproteobacteria	Salinisphaerales	Solimonadaceae		Sed DOM
FW305-C-283	MN067565	93.5	Proteobacteria	Gammaproteobacteria	Salinisphaerales	Solimonadaceae		Sed DOM
FW305-C-285	MN067567	93.5	Proteobacteria	Gammaproteobacteria	Salinisphaerales	Solimonadaceae		Cell lysate
FW305-C-30-12	MT160410	93.4	Proteobacteria	Gammaproteobacteria	Salinisphaerales	Solimonadaceae		Cell lysate
FW305-C-3	MK402977	94.6	Proteobacteria	Gammaproteobacteria	Salinisphaerales	Solimonadaceae		Sed DOM
FW305-C-252	MK559504	99.8	Proteobacteria	Gammaproteobacteria	Xanthomonadales	Xanthomonadaceae	Stenotrophomonas	Cell lysate

Table S5. Taxonomy of selected (one-way ANOVA with Dunnett's multiple comparison test, p-value < 0.01) OTUs that were significantly enriched in simple or complex C-amended enrichments compared to the unamended control, corresponding with OTUs illustrated in Figure 4A. Taxonomy was assigned using the SILVA database. Absence of an identified taxonomic level indicates no match with confidence > 0.5 returned from the SILVA database. Representative isolates (99–100% identity) obtained in this study are also included.

OTU	Kingdom_SILVA	Phylum_SILVA	Class_SILVA	Order_SILVA	Family_SILVA	Genus_SILVA	Enriched C type	Enriched condition*	Isolate ID (per BLAST)	Identity (between amplicon and isolate 16S sequence)
denovo713	Bacteria	Acidobacteriota	Acidobacteriae	Bryobacterales	Bryobacteraceae	Bryobacter	Ex complex C	H10,H20,H30		
denovo778	Bacteria	Acidobacteriota	Acidobacteriae	Paludibaculum			Ex complex C	H10,H20,H30		
denovo2868	Bacteria	Acidobacteriota	Acidobacteriae	Paludibaculum			Ex complex C	H10,H20,H30		
denovo1831	Bacteria	Acidobacteriota	Blastocatellia	44159			Ex simple C	C30		
denovo3145	Bacteria	Acidobacteriota	Blastocatellia	44159			Ex simple C	C30		
denovo605	Bacteria	Acidobacteriota	Vicinamibacteria	Vicinamibacterales			Ex complex C	H20,H30		
denovo1267	Bacteria	Acidobacteriota	Vicinamibacteria	Vicinamibacterales	Vicinamibacteraceae		Ex complex C	G20,G30,H20,H30		
denovo973	Bacteria	Acidobacteriota	Acidobacteriae				Ex complex C	H30		
denovo2461	Bacteria	Actinobacteriota	Acidimicrobiia	Microtrichales	Ilumatobacteraceae		Ex complex C	H30		
denovo110	Bacteria	Actinobacteriota	Actinobacteria	Micrococcales	Intrasporangiaceae	Phycicoccus	Ex complex C	G10,G30		
denovo2834	Bacteria	Actinobacteriota	Actinobacteria	Corynebacteriales	Mycobacteriaceae	Mycobacterium	Ex complex C	G10,G20		
denovo243	Bacteria	Actinobacteriota	Actinobacteria	Micrococcales	Microbacteriaceae	Microbacterium	Ex complex C	G10,G20,H30	FW305-C-20	100%
denovo422	Bacteria	Actinobacteriota	Actinobacteria	Micrococcales	Micrococcaceae	Paenarthrobacter	Ex complex C	H10,H20,H30	FW305-C-256,257	100%
denovo61	Bacteria	Actinobacteriota	Actinobacteria	Propionibacteriales	Nocardioidaceae	Nocardioides	Ex complex C	G10		
denovo226	Bacteria	Actinobacteriota	Actinobacteria	Propionibacteriales	Nocardioidaceae	Nocardioides	Ex complex C	G20,H20		
denovo2471	Bacteria	Armatimonadota	Armatimonadia	Armatimonadales			Ex complex C	H10		
denovo1843	Bacteria	Armatimonadota	Chthonomonadetes	Chthonomonadales	Chthonomonadaceae	Chthonomonas	Ex complex C	H20,H30		
denovo1800	Bacteria	Armatimonadota	Fimbriimonadia	Fimbriimonadales	Fimbriimonadaceae		Ex complex C	H30		
denovo3318	Bacteria	Armatimonadota	uncultured				Ex complex C	H20		
denovo392	Bacteria	Bacteroidota	Bacteroidia	Chitinophagales	Chitinophagaceae	Edaphobaculum	Ex complex C	H30		
denovo756	Bacteria	Bacteroidota	Bacteroidia	Chitinophagales	Chitinophagaceae	Edaphobaculum	Ex complex C	H10,H30		
denovo526	Bacteria	Bacteroidota	Bacteroidia	Chitinophagales	Chitinophagaceae		Both complex and simple C	C20,G10,H10	FW305-C-185	100%
denovo614	Bacteria	Bacteroidota	Bacteroidia	Chitinophagales	Chitinophagaceae		Both complex and simple C	C20,E30,G10,H10		
denovo1852	Bacteria	Bacteroidota	Bacteroidia	Chitinophagales	Chitinophagaceae		Ex simple C	A30		
denovo1938	Bacteria	Bacteroidota	Bacteroidia	Chitinophagales	Chitinophagaceae		Ex complex C	H10,H20		
denovo3451	Bacteria	Bacteroidota	Bacteroidia	Chitinophagales	Chitinophagaceae		Ex simple C	A10		
denovo158	Bacteria	Bacteroidota	Bacteroidia	Chitinophagales	Saprospiraceae	Phaeodactylibacter	Ex complex C	H10,H20,H30		
denovo453	Bacteria	Bacteroidota	Bacteroidia	Chitinophagales	Saprospiraceae	uncultured	Ex complex C	H20,H30		
denovo994	Bacteria	Bacteroidota	Bacteroidia	Cytophagales	Spirosomaceae	Emticicia	Ex complex C	H10	FW305-C-80,30-33	100%
denovo1165	Bacteria	Bacteroidota	Bacteroidia	Cytophagales	Spirosomaceae	Arcicella	Ex simple C	A10,A20		
denovo2050	Bacteria	Bacteroidota	Bacteroidia	Cytophagales	Spirosomaceae	Runella	Both complex and simple C	C30,G10,H10		
denovo271	Bacteria	Bacteroidota	Bacteroidia	Cytophagales	Spirosomaceae		Ex simple C	A30		
denovo2606	Bacteria	Bacteroidota	Bacteroidia	Cytophagales	Microscillaceae		Ex simple C	A30		
denovo2744	Bacteria	Bacteroidota	Bacteroidia	Cytophagales	Microscillaceae	uncultured	Ex complex C	H30		
denovo3163	Bacteria	Bacteroidota	Bacteroidia	Cytophagales	Spirosomaceae	Flectobacillus	Ex simple C	A10,A20,A30,B20,B30	FW305-C-70,79,258,263,264,265,293	100%
denovo2749	Bacteria	Bacteroidota	Bacteroidia	Flavobacteriales	Crocinitomicaceae	Fluviicola	Ex complex C	G10,G30,H20,H30		
denovo1317	Bacteria	Bacteroidota	Bacteroidia	Flavobacteriales	Flavobacteriaceae	Flavobacterium	Ex complex C	H10		
denovo2523	Bacteria	Bacteroidota	Bacteroidia	Flavobacteriales	Flavobacteriaceae	Flavobacterium	Ex complex C	H10	FW305-C-10-5	100%
denovo2119	Bacteria	Bacteroidota	Bacteroidia	Flavobacteriales	Weeksellaceae	Chryseobacterium	Ex complex C	G10		
denovo1239	Bacteria	Bacteroidota	Bacteroidia	Sphingobacteriales	Sphingobacteriaceae	Pedobacter	Ex complex C	H10	FW305-C-75,78,277	100%

denovo1361	Bacteria	Bacteroidota	Bacteroidia	Sphingobacteriales	Sphingobacteriaceae	Pedobacter	Ex complex C	G20,G30,H10,H30		
denovo1405	Bacteria	Bacteroidota	Bacteroidia	Sphingobacteriales	env.OPS 17		Ex complex C	G10	FW305-C-21,23	100%
denovo2797	Bacteria	Bacteroidota	Bacteroidia	Sphingobacteriales	env.OPS 17		Ex complex C	H20,H30		
denovo3006	Bacteria	Bacteroidota	Bacteroidia	Sphingobacteriales	NS11-12	marine group	Ex complex C	H10		
denovo3081	Bacteria	Bacteroidota	Bacteroidia	Sphingobacteriales	NS11-12	marine group	Ex complex C	H10,H30		
denovo2736	Bacteria	Bacteroidota	Kapabacteria	Kapabacteriales			Ex simple C	B10,B20,C20,C30		
denovo1248	Bacteria	Chloroflexi	Anaerolineae	Caldilineales	Caldilineaceae	uncultured	Ex complex C	H20,H30		
denovo2103	Bacteria	Firmicutes	Bacilli	Paenibacillales	Paenibacillaceae	Paenibacillus	Ex complex C	G20		
denovo235	Bacteria	Myxococcota	Myxococcia	Myxococcales	Myxococcaceae	P3OB-42	Ex simple C	B10,B20,B30		
denovo1153	Bacteria	Myxococcota	Polyangia	Blfdi19			Ex complex C	G30,H20		
denovo1794	Bacteria	Myxococcota	Polyangia	mle1-27			Ex complex C	G10		
denovo2775	Bacteria	Myxococcota	Polyangia	Polyangiales	Polyangiaceae	Pajaroellobacter	Ex complex C	G20		
denovo2589	Bacteria	Nitrospirota	Nitrospiria	Nitrospirales	Nitrospiraceae	Nitrospira	Ex complex C	G20,G30,H20,H30		
denovo2235	Bacteria	Planctomycetota	Phycisphaerae	Phycisphaerales	Phycisphaeraceae	SM1A02	Both complex and simple C	C30,G20,G30		
denovo2280	Bacteria	Planctomycetota	Planctomycetes	Gemmatales	Gemmataceae	Fimbriiglobus	Ex complex C	H30		
denovo2118	Bacteria	Planctomycetota	Planctomycetes	Gemmatales	Gemmataceae	Gemmata	Ex complex C	H30		
denovo3111	Bacteria	Planctomycetota	Planctomycetes	Gemmatales	Gemmataceae	Gemmata	Ex complex C	H30		
denovo1115	Bacteria	Planctomycetota	Planctomycetes	Gemmatales	Gemmataceae	uncultured	Ex complex C	H10,H20,H30		
denovo252	Bacteria	Planctomycetota	Planctomycetes	Gemmatales	Gemmataceae		Ex complex C	H10,H20		
denovo1257	Bacteria	Planctomycetota	Planctomycetes	Isosphaerales	Isosphaeraceae		Ex complex C	H30		
denovo2264	Bacteria	Planctomycetota	Planctomycetes	Isosphaerales	Isosphaeraceae		Ex complex C	H30		
denovo240	Bacteria	Planctomycetota	Planctomycetes	Pirellulales	Pirellulaceae	uncultured	Ex complex C	H30		
denovo2404	Bacteria	Planctomycetota	Planctomycetes	Pirellulales	Pirellulaceae	uncultured	Ex complex C	G30		
denovo3447	Bacteria	Planctomycetota	Planctomycetes	Planctomycetales	Rubinisphaeraceae	Planctomicrobium	Ex complex C	H10,H20,H30		
denovo358	Bacteria	Planctomycetota	Planctomycetes	Planctomycetales	Schlesneriaceae	Planctopirus	Ex complex C	H30		
denovo3048	Bacteria	Planctomycetota	Planctomycetes	Planctomycetales	Schlesneriaceae	Schlesneria	Ex complex C	G10,G20,G30		
denovo826	Bacteria	Planctomycetota	Planctomycetes	Planctomycetales	uncultured		Ex complex C	H30		
denovo2796	Bacteria	Planctomycetota	Planctomycetes	Planctomycetales	uncultured		Ex complex C	H20,H30		
denovo3219	Bacteria	Planctomycetota	Planctomycetes	Planctomycetales	uncultured		Ex complex C	H30		
denovo2577	Bacteria	Planctomycetota	Planctomycetes	Planctomycetales			Ex complex C	H30		
denovo141	Bacteria	Planctomycetota	Planctomycetes	uncultured			Ex complex C	G30		
denovo3050	Bacteria	Proteobacteria	Alphaproteobacteria	Acetobacterales	Acetobacteraceae	Roseococcus	Ex simple C	E30	FW305-C-136D	100%
denovo1050	Bacteria	Proteobacteria	Alphaproteobacteria	Acetobacterales	Acetobacteraceae	Roseomonas	Ex complex C	H30		
denovo2454	Bacteria	Proteobacteria	Alphaproteobacteria	Acetobacterales	Acetobacteraceae	uncultured	Ex complex C	H20,H30		
denovo1593	Bacteria	Proteobacteria	Alphaproteobacteria	Acetobacterales	Acetobacteraceae		Ex complex C	H10,H20,H30		
denovo2739	Bacteria	Proteobacteria	Alphaproteobacteria	Azospirillales	Azospirillaceae	Azospirillum	Ex complex C	G10,G20,G30		
denovo375	Bacteria	Proteobacteria	Alphaproteobacteria	Azospirillales	Azospirillaceae		Ex complex C	H30		
denovo2423	Bacteria	Proteobacteria	Alphaproteobacteria	Azospirillales	Inquilinaceae	Inquilinus	Ex complex C	H20,H30		
denovo310	Bacteria	Proteobacteria	Alphaproteobacteria	Caulobacterales	Caulobacteraceae	Brevundimonas	Ex simple C	B20,B30,E30	FW305-C-18	99%
denovo391	Bacteria	Proteobacteria	Alphaproteobacteria	Caulobacterales	Caulobacteraceae	Phenylobacterium	Ex simple C	B20,E20,E30		
denovo1773	Bacteria	Proteobacteria	Alphaproteobacteria	Caulobacterales	Caulobacteraceae	Caulobacter	Both complex and simple C	C30,H10,H20,H30	FW305-C-128	100%
denovo1803	Bacteria	Proteobacteria	Alphaproteobacteria	Caulobacterales	Caulobacteraceae	Caulobacter	Ex simple C	A10,A20,A30,B10		
denovo1897	Bacteria	Proteobacteria	Alphaproteobacteria	Caulobacterales	Caulobacteraceae		Ex simple C	E20,E30		
denovo1985	Bacteria	Proteobacteria	Alphaproteobacteria	Caulobacterales	Caulobacteraceae		Ex simple C	E30		
denovo2422	Bacteria	Proteobacteria	Alphaproteobacteria	Caulobacterales	Caulobacteraceae		Ex simple C	B20,B30		

denovo564	Bacteria	Proteobacteria	Alphaproteobacteria	Caulobacterales	Hyphomonadaceae	Hirschia	Both complex and simple C	C30,H10,H20,H30		
denovo373	Bacteria	Proteobacteria	Alphaproteobacteria	Paracaedibacterales	Paracaedibacteraceae	uncultured	Ex complex C	G10		
denovo448	Bacteria	Proteobacteria	Alphaproteobacteria	Reyranellales	Reyranellaceae	Reyranella	Ex complex C	G30,H10,H20,H30		
denovo657	Bacteria	Proteobacteria	Alphaproteobacteria	Reyranellales	Reyranellaceae	Reyranella	Ex complex C	G30,H20,H30		
denovo1557	Bacteria	Proteobacteria	Alphaproteobacteria	Reyranellales	Reyranellaceae	Reyranella	Both complex and simple C	C30,H10,H20,H30	FW305-C-30-S3,30-S5,30-S8,30-S9,30-S10	99%
denovo2345	Bacteria	Proteobacteria	Alphaproteobacteria	Reyranellales	Reyranellaceae	uncultured	Ex complex C	H30		
denovo1783	Bacteria	Proteobacteria	Alphaproteobacteria	Rhizobiales	A0839		Ex complex C	H30		
denovo1081	Bacteria	Proteobacteria	Alphaproteobacteria	Rhizobiales	Beijerinckiaceae	Bosea	Ex simple C	C30		
denovo2324	Bacteria	Proteobacteria	Alphaproteobacteria	Rhizobiales	Beijerinckiaceae	Bosea	Both complex and simple C	C30,H10	FW305-C-101	100%
denovo2653	Bacteria	Proteobacteria	Alphaproteobacteria	Rhizobiales	Beijerinckiaceae	Bosea	Both complex and simple C	B20,B30,C20,C30,E30,G10,H10	FW305-C-74,92,116,117,121,275	100%
denovo1103	Bacteria	Proteobacteria	Alphaproteobacteria	Rhizobiales	Beijerinckiaceae		Ex complex C	H20,H30		
denovo3223	Bacteria	Proteobacteria	Alphaproteobacteria	Rhizobiales	Beijerinckiaceae	Methylobacterium-Methylorubrum	Ex simple C	B10	FW305-C-198	100%
denovo3071	Bacteria	Proteobacteria	Alphaproteobacteria	Rhizobiales	Devosiaceae	Devosia	Ex complex C	G30,H10,H20,H30	FW305-C-47,77,91,93,99,106,123,141,156	100%
denovo1805	Bacteria	Proteobacteria	Alphaproteobacteria	Rhizobiales	Hyphomicrobiaceae		Ex complex C	H20,H30		
denovo1807	Bacteria	Proteobacteria	Alphaproteobacteria	Rhizobiales	Hyphomicrobiaceae	Hyphomicrobium	Ex complex C	H20		
denovo1951	Bacteria	Proteobacteria	Alphaproteobacteria	Rhizobiales	Hyphomicrobiaceae	Pedomicrobium	Ex simple C	A20,A30,B20,C20,C30		
denovo3143	Bacteria	Proteobacteria	Alphaproteobacteria	Rhizobiales	Kaistiaceae	Kaistia	Ex complex C	H10,H20,H30		
denovo1745	Bacteria	Proteobacteria	Alphaproteobacteria	Rhizobiales	Rhizobiaceae	Allorhizobium-Neorhizobium-Pararhizobium-Rhizobium	Ex complex C	G10,G20,G30,H10,H20	FW305-C-176I	100%
denovo3150	Bacteria	Proteobacteria	Alphaproteobacteria	Rhizobiales	Rhizobiaceae	Allorhizobium-Neorhizobium-Pararhizobium-Rhizobium	Ex complex C	G30	FW305-C-8,26,27,30,39,43	100%
denovo388	Bacteria	Proteobacteria	Alphaproteobacteria	Rhizobiales	Rhizobiales	Incertae Sedis uncultured	Ex complex C	G10,H20,H30		
denovo736	Bacteria	Proteobacteria	Alphaproteobacteria	Rhizobiales	Rhizobiales	Incertae Sedis Phreatobacter	Both complex and simple C	C30,G20,H10	FW305-C-134A	100%
denovo2687	Bacteria	Proteobacteria	Alphaproteobacteria	Rhizobiales	Rhizobiales	Incertae Sedis Nordella	Ex complex C	G10,G20,G30	FW305-C-112	100%
denovo1507	Bacteria	Proteobacteria	Alphaproteobacteria	Rhizobiales	Xanthobacteraceae	Xanthobacter	Both complex and simple C	C30,H10,H20,H30		
denovo2031	Bacteria	Proteobacteria	Alphaproteobacteria	Rhizobiales	Xanthobacteraceae	Xanthobacter	Ex complex C	H10,H20,H30		
denovo2630	Bacteria	Proteobacteria	Alphaproteobacteria	Rhizobiales	Xanthobacteraceae		Ex complex C	H20		
denovo568	Bacteria	Proteobacteria	Alphaproteobacteria	Rhizobiales			Ex complex C	H20,H30		
denovo1251	Bacteria	Proteobacteria	Alphaproteobacteria	Rhizobiales			Ex complex C	G10,G20,G30,H10,H20,H30		
denovo1767	Bacteria	Proteobacteria	Alphaproteobacteria	Rhizobiales			Ex complex C	H20,H30		
denovo2865	Bacteria	Proteobacteria	Alphaproteobacteria	Rhizobiales			Ex complex C	H20,H30		
denovo2956	Bacteria	Proteobacteria	Alphaproteobacteria	Rhizobiales			Ex complex C	G20,G30,H10,H20,H30		
denovo3101	Bacteria	Proteobacteria	Alphaproteobacteria	Rhizobiales			Ex complex C	H20		
denovo3203	Bacteria	Proteobacteria	Alphaproteobacteria	Rhizobiales			Ex complex C	H10,H20,H30		
denovo1039	Bacteria	Proteobacteria	Alphaproteobacteria	Rhodobacterales	Rhodobacteraceae	Rhodobacter	Ex complex C	H20		
denovo338	Bacteria	Proteobacteria	Alphaproteobacteria	Rhodospirillales	Rhodospirillaceae	uncultured	Ex complex C	G10		

denovo1646	Bacteria	Proteobacteria	Alphaproteobacteria	Rhodospirillales	Rhodospirillaceae	uncultured	Ex complex C	H30		
denovo2723	Bacteria	Proteobacteria	Alphaproteobacteria	Sphingomonadales	Sphingomonadaceae	Sphingoaurantiacus	Ex complex C	H20,H30		
denovo537	Bacteria	Proteobacteria	Alphaproteobacteria	Sphingomonadales	Sphingomonadaceae	Sphingomonas	Ex complex C	G20,G30		
denovo1760	Bacteria	Proteobacteria	Alphaproteobacteria	Sphingomonadales	Sphingomonadaceae	Sphingomonas	Ex complex C	H10,H20,H30		
denovo2244	Bacteria	Proteobacteria	Alphaproteobacteria	Sphingomonadales	Sphingomonadaceae	Sphingomonas	Ex simple C	A20,A30,B20,B30	FW305-C-94	99%
denovo1809	Bacteria	Proteobacteria	Alphaproteobacteria	Sphingomonadales	Sphingomonadaceae	Sphingopyxis	Ex complex C	H20,H30	FW305-C-54	100%
denovo2538	Bacteria	Proteobacteria	Alphaproteobacteria	Sphingomonadales	Sphingomonadaceae	Novosphingobium	Ex simple C	B10		
denovo469	Bacteria	Proteobacteria	Alphaproteobacteria	Sphingomonadales	Sphingomonadaceae	uncultured	Ex simple C	C30		
denovo3122	Bacteria	Proteobacteria	Alphaproteobacteria	Sphingomonadales	Sphingomonadaceae		Ex simple C	B20,C30		
denovo3441	Bacteria	Proteobacteria	Alphaproteobacteria	Sphingomonadales	Sphingomonadaceae		Ex complex C	H10,H20,H30		
denovo1741	Bacteria	Proteobacteria	Alphaproteobacteria				Ex complex C	H10		
denovo2076	Bacteria	Proteobacteria	Alphaproteobacteria				Ex complex C	G30		
denovo2322	Bacteria	Proteobacteria	Alphaproteobacteria				Ex complex C	H10,H20,H30		
denovo1156	Bacteria	Proteobacteria	Gammaproteobacteria	Burkholderiales	Alcaligenaceae	Achromobacter	Ex complex C	G30,H10,H20,H30	FW305-C-114,107,13,287,31	100%
denovo2145	Bacteria	Proteobacteria	Gammaproteobacteria	Burkholderiales	Comamonadaceae	Curvibacter	Ex simple C	E20,E30	FW305-C-25	99%
denovo411	Bacteria	Proteobacteria	Gammaproteobacteria	Burkholderiales	Comamonadaceae		Ex simple C	C20,C30	FW305-C-10-12,10-24,24,29,41,83,90,268	100%
denovo1078	Bacteria	Proteobacteria	Gammaproteobacteria	Burkholderiales	Comamonadaceae		Ex simple C	B10	FW305-C-20-26	99%
denovo2249	Bacteria	Proteobacteria	Gammaproteobacteria	Burkholderiales	Comamonadaceae		Ex complex C	G30		
denovo3152	Bacteria	Proteobacteria	Gammaproteobacteria	Burkholderiales	Comamonadaceae		Ex simple C	C30		
denovo194	Bacteria	Proteobacteria	Gammaproteobacteria	Burkholderiales	Nitrosomonadaceae	Nitrosomonas	Ex complex C	G10,G20		
denovo2498	Bacteria	Proteobacteria	Gammaproteobacteria	Burkholderiales	Nitrosomonadaceae	MND1	Ex complex C	G20,G30		
denovo2818	Bacteria	Proteobacteria	Gammaproteobacteria	Burkholderiales	Nitrosomonadaceae		Ex complex C	H20,H30		
denovo1112	Bacteria	Proteobacteria	Gammaproteobacteria	Burkholderiales	Oxalobacteraceae	Duganella	Ex simple C	E10,E20		
denovo1480	Bacteria	Proteobacteria	Gammaproteobacteria	Burkholderiales	Oxalobacteraceae	Duganella	Ex simple C	E10		
denovo3204	Bacteria	Proteobacteria	Gammaproteobacteria	Burkholderiales	Oxalobacteraceae		Ex simple C	E10,E20		
denovo273	Bacteria	Proteobacteria	Gammaproteobacteria	Burkholderiales	Rhodocyclaceae	Ferribacterium	Ex simple C	E20		
denovo489	Bacteria	Proteobacteria	Gammaproteobacteria	Burkholderiales	Rhodocyclaceae	Dechloromonas	Ex simple C	E20		
denovo812	Bacteria	Proteobacteria	Gammaproteobacteria	Burkholderiales	Rhodocyclaceae	Dechloromonas	Both complex and simple C	E20,G10,G20	FW305-C-19	100%
denovo1834	Bacteria	Proteobacteria	Gammaproteobacteria	Burkholderiales	Rhodocyclaceae		Both complex and simple C	C10,C20,C30,G30,H20,H30		
denovo2566	Bacteria	Proteobacteria	Gammaproteobacteria	Burkholderiales	Rhodocyclaceae		Ex simple C	E10,E20,E30		
denovo662	Bacteria	Proteobacteria	Gammaproteobacteria	Burkholderiales	TRA3-20		Ex complex C	G20		
denovo2380	Bacteria	Proteobacteria	Gammaproteobacteria	Burkholderiales	TRA3-20		Ex complex C	H20		
denovo2496	Bacteria	Proteobacteria	Gammaproteobacteria	Enterobacterales	Enterobacteriaceae	Escherichia-Shigella	Ex simple C	A20		
denovo326	Bacteria	Proteobacteria	Gammaproteobacteria	Legionellales	Legionellaceae	Legionella	Both complex and simple C	C30,G10,G30		
denovo2451	Bacteria	Proteobacteria	Gammaproteobacteria	Legionellales	Legionellaceae	Legionella	Ex complex C	H10,H20,H30		
denovo2504	Bacteria	Proteobacteria	Gammaproteobacteria	Legionellales	Legionellaceae	Legionella	Ex complex C	H20,H30		
denovo1790	Bacteria	Proteobacteria	Gammaproteobacteria	Pseudomonadales	Moraxellaceae	[Agitococcus] lubricus group	Ex simple C	E10		
denovo2793	Bacteria	Proteobacteria	Gammaproteobacteria	Pseudomonadales	Moraxellaceae	Alkanindiges	Ex simple C	E10		
denovo1079	Bacteria	Proteobacteria	Gammaproteobacteria	Pseudomonadales	Pseudomonadaceae	Pseudomonas	Ex simple C	E30		
denovo2567	Bacteria	Proteobacteria	Gammaproteobacteria	Pseudomonadales	Pseudomonadaceae	Pseudomonas	Both complex and simple C	E30,G10	FW305-C-5,12	100%
denovo3272	Bacteria	Proteobacteria	Gammaproteobacteria	Salinisphaerales	Solimonadaceae		Ex simple C	E10		
denovo1431	Bacteria	Proteobacteria	Gammaproteobacteria				Ex simple C	B20,B30		

denovo1229	Bacteria	Verrucomicrobiota	Verrucomicrobiae	Opitutales	Opitutaceae	Lacunisphaera	Ex complex C	G10,G20,G30		
denovo2924	Bacteria	Verrucomicrobiota	Verrucomicrobiae	Pedosphaerales	Pedosphaeraceae	SH3-11	Ex complex C	H10,H20		
denovo2995	Bacteria	Verrucomicrobiota	Verrucomicrobiae	Pedosphaerales	Pedosphaeraceae		Ex complex C	G20,G30		
denovo743	Bacteria	Verrucomicrobiota	Verrucomicrobiae	Verrucomicrobiales	Verrucomicrobiaceae	Prosthecobacter	Ex complex C	H10		
denovo2188	Bacteria	WPS-2					Ex simple C	E30		
*A: glucose; B: Acetate; C: Benzoate; E: Oleic acid; G: Cell lysate; H: Sediment DOM; 10/20/30: enriched on day10/20/30										

Table S6. The list of isolates representing 56 distinct bacterial species obtained in this study, corresponding with isolates illustrated in Figure 5.

Isolate ID	Accession number	Identity	Phylum	Class	Order	Family	Genus	Relative abundance (%) of matched (e-value < 1E-100) OTU in the Initial sample
FW305-C-125	MK402928	99.1	Actinobacteria	Actinobacteria	Micrococcales	Microbacteriaceae	Herbiconiux	3.15
FW305-C-176A	MK402930	99.4	Actinobacteria	Actinobacteria	Micrococcales	Microbacteriaceae	Leucobacter	0.69
FW305-C-20-19	MT160389	99.2	Actinobacteria	Actinobacteria	Micrococcales	Microbacteriaceae	Microbacterium	0.69
FW305-C-179A	MN067593	98.8	Actinobacteria	Actinobacteria	Micrococcales	Microbacteriaceae		0.69
FW305-C-20-9	MT210153	99.8	Actinobacteria	Actinobacteria	Micrococcales	Micrococcaceae	Micrococcus	0.69
FW305-C-261	MK559506	99.8	Actinobacteria	Actinobacteria	Micrococcales	Micrococcaceae		0.69
FW305-C-256	MK559505	100.0	Actinobacteria	Actinobacteria	Micrococcales	Micrococcaceae	Paenarthrobacter	0.69
FW305-C-61	MN067618	98.6	Bacteroidetes	Bacteroidia	Chitinophagales	Chitinophagaceae	Sediminibacterium	5.05
FW305-C-269	MK559507	96.1	Bacteroidetes	Bacteroidia	Chitinophagales	Chitinophagaceae	Sediminibacterium	0.86
FW305-C-185	MK402934	97.9	Bacteroidetes	Bacteroidia	Chitinophagales	Chitinophagaceae	Terrimonas	0.27
FW305-C-84	MK402937	94.1	Bacteroidetes	Bacteroidia	Cytophagales	Spirosomaceae	Dyadobacter	< 0.01
FW305-C-80	MK402936	97.9	Bacteroidetes	Bacteroidia	Cytophagales	Spirosomaceae	Emticicia	< 0.01
FW305-C-70	MK402935	99.0	Bacteroidetes	Bacteroidia	Cytophagales	Spirosomaceae	Flectobacillus	< 0.01
FW305-C-20-21	MT160392	98.1	Bacteroidetes	Bacteroidia	Cytophagales	Spirosomaceae	Spirosoma	< 0.01
FW305-C-10-5	MT160391	96.5	Bacteroidetes	Bacteroidia	Flavobacteriales	Flavobacteriaceae	Flavobacterium	< 0.01
FW305-C-21	MK402939	96.3	Bacteroidetes	Bacteroidia	Sphingobacteriales	env.OPS 17		< 0.01
FW305-C-277	MN067579	97.7	Bacteroidetes	Bacteroidia	Sphingobacteriales	Sphingobacteriaceae	Pedobacter	< 0.01
FW305-C-10-2	MT160394	100.0	Deinococcota	Deinococci	Deinococcales	Deinococcaceae	Deinococcus	< 0.01
FW305-C-191	MK402940	99.6	Firmicutes	Bacilli	Bacillales	Bacillaceae	Bacillus	< 0.01
FW305-C-190	MK402943	99.3	Firmicutes	Bacilli	Bacillales	Paenibacillaceae	Paenibacillus	< 0.01
FW305-C-202	MK402944	99.7	Firmicutes	Bacilli	Bacillales	Paenibacillaceae	Paenibacillus	< 0.01
FW305-C-11	MN067603	99.9	Firmicutes	Bacilli	Brevibacillales	Brevibacillaceae	Brevibacillus	< 0.01
FW305-C-103	MK402958	98.0	Proteobacteria	Alphaproteobacteria	Acetobacterales	Acetobacteraceae	Roseomonas	< 0.01
FW305-C-119	MK402959	97.5	Proteobacteria	Alphaproteobacteria	Acetobacterales	Acetobacteraceae	Roseomonas	< 0.01
FW305-C-136D	MN305721	95.8	Proteobacteria	Alphaproteobacteria	Acetobacterales	Acetobacteraceae	Roseococcus	< 0.01
FW305-C-18	MK402945	100.0	Proteobacteria	Alphaproteobacteria	Caulobacterales	Caulobacteraceae	Brevundimonas	0.85
FW305-C-128	MK402946	99.5	Proteobacteria	Alphaproteobacteria	Caulobacterales	Caulobacteraceae	Caulobacter	0.85
FW305-C-130	MK402947	98.3	Proteobacteria	Alphaproteobacteria	Caulobacterales	Caulobacteraceae	Caulobacter	3.37
FW305-C-30-S1	MT538329	98.1	Proteobacteria	Alphaproteobacteria	Caulobacterales	Caulobacteraceae	Phenylobacterium	0.56
FW305-C-30-S3	MT538330	98.9	Proteobacteria	Alphaproteobacteria	Reyranellales	Reyranellaceae	Reyranella	< 0.01

FW305-C-101	MK402951	99.7	Proteobacteria	Alphaproteobacteria	Rhizobiales	Beijerinckiaceae	Bosea	0.61
FW305-C-198	MK402954	99.7	Proteobacteria	Alphaproteobacteria	Rhizobiales	Beijerinckiaceae	Methylobacterium-Methylorubrum	< 0.01
FW305-C-149	MN067659	98.8	Proteobacteria	Alphaproteobacteria	Rhizobiales	Devosiaceae	Devosia	0.60
FW305-C-176I	MK402957	99.5	Proteobacteria	Alphaproteobacteria	Rhizobiales	Rhizobiaceae	Allorhizobium-Neorhizobium-Pararhizobium-Rhizobium	0.60
FW305-C-52	MK402949	100.0	Proteobacteria	Alphaproteobacteria	Rhizobiales	Rhizobiaceae	Aminobacter	2.25
FW305-C-10-9	MT160397	99.7	Proteobacteria	Alphaproteobacteria	Rhizobiales	Rhizobiaceae	Ensifer	2.25
FW305-C-20-14	MT160398	98.9	Proteobacteria	Alphaproteobacteria	Rhizobiales	Rhizobiaceae	Shinella	0.60
FW305-C-8	MK402956	95.9	Proteobacteria	Alphaproteobacteria	Rhizobiales	Rhizobiaceae		2.25
FW305-C-134A	MK402966	98.4	Proteobacteria	Alphaproteobacteria	Rhizobiales	Rhizobiales-Incertae-Sedis	Phreatobacter	0.60
FW305-C-112	MK402955	99.8	Proteobacteria	Alphaproteobacteria	Rhizobiales	Rhizobiales-Incertae-Sedis	Nordella	0.60
FW305-C-122	MK402948	99.6	Proteobacteria	Alphaproteobacteria	Rhizobiales	Xanthobacteraceae	Afipia	0.61
FW305-C-111	MK402961	98.7	Proteobacteria	Alphaproteobacteria	Sphingomonadales	Sphingomonadaceae	Novosphingobium	1.05
FW305-C-56	MK402964	99.3	Proteobacteria	Alphaproteobacteria	Sphingomonadales	Sphingomonadaceae	Sphingomonas	1.16
FW305-C-20-25	MT160401	98.5	Proteobacteria	Alphaproteobacteria	Sphingomonadales	Sphingomonadaceae	Sphingopyxis	1.05
FW305-C-13	MN067605	99.9	Proteobacteria	Gammaproteobacteria	Burkholderiales	Alcaligenaceae		2.03
FW305-C-176C	MK402970	99.6	Proteobacteria	Gammaproteobacteria	Burkholderiales	Burkholderiaceae	Cupriavidus	0.32
FW305-C-20-26	MT160404	99.6	Proteobacteria	Gammaproteobacteria	Burkholderiales	Comamonadaceae	Acidovorax	0.59
FW305-C-7	MK402973	100.0	Proteobacteria	Gammaproteobacteria	Burkholderiales	Comamonadaceae	Variovorax	0.59
FW305-C-24	MK402972	99.9	Proteobacteria	Gammaproteobacteria	Burkholderiales	Comamonadaceae		0.93
FW305-C-294	MK559509	98.5	Proteobacteria	Gammaproteobacteria	Burkholderiales	Comamonadaceae		0.59
FW305-C-19	MK402974	99.9	Proteobacteria	Gammaproteobacteria	Burkholderiales	Rhodocyclaceae	Dechloromonas	0.30
FW305-C-5	MK402978	100.0	Proteobacteria	Gammaproteobacteria	Pseudomonadales	Pseudomonadaceae	Pseudomonas	1.30
FW305-C-57	MN067614	99.1	Proteobacteria	Gammaproteobacteria	Salinisphaerales	Solimonadaceae	Hydrocarboniphaga	0.80
FW305-C-2	MK402976	93.3	Proteobacteria	Gammaproteobacteria	Salinisphaerales	Solimonadaceae		0.80
FW305-C-3	MK402977	94.6	Proteobacteria	Gammaproteobacteria	Salinisphaerales	Solimonadaceae		0.80
FW305-C-252	MK559504	99.8	Proteobacteria	Gammaproteobacteria	Xanthomonadales	Xanthomonadaceae	Stenotrophomonas	0.80
